# Zn-ion ultrafluidity via bioinspired ion channel for ultralong lifespan Zn-ion battery

**DOI:** 10.1093/nsr/nwae199

**Published:** 2024-06-12

**Authors:** Fan Zhang, Ting Liao, Dong-Chen Qi, Tony Wang, Yanan Xu, Wei Luo, Cheng Yan, Lei Jiang, Ziqi Sun

**Affiliations:** School of Chemistry and Physics, Queensland University of Technology, Brisbane 4000, Australia; School of Mechanical Medical and Process Engineering, Queensland University of Technology, Brisbane 4000, Australia; School of Chemistry and Physics, Queensland University of Technology, Brisbane 4000, Australia; Centre for Materials Science, Queensland University of Technology, Brisbane 4000, Australia; Central Analytical Research Facility, Queensland University of Technology, Brisbane 4000, Australia; Centre for Materials Science, Queensland University of Technology, Brisbane 4000, Australia; Central Analytical Research Facility, Queensland University of Technology, Brisbane 4000, Australia; Centre for Materials Science, Queensland University of Technology, Brisbane 4000, Australia; State Key Laboratory for Modification of Chemical Fibers and Polymer Materials, College of Materials Science and Engineering, Donghua University, Shanghai 201620, China; School of Mechanical Medical and Process Engineering, Queensland University of Technology, Brisbane 4000, Australia; Key Laboratory of Bio-inspired Materials and Interfacial Science, Technical Institute of Physics and Chemistry, Chinese Academy of Sciences, Beijing 100190, China; School of Mathematical and Physical Sciences, University of Technology Sydney, Sydney 2007, Australia; School of Chemistry and Physics, Queensland University of Technology, Brisbane 4000, Australia

**Keywords:** aqueous Zn-ion battery, ultrafluidic, bioinspired electrode, dendrite-free Zn deposition

## Abstract

Rechargeable aqueous Zn-ion batteries have been deemed a promising energy storage device. However, the dendrite growth and side reactions have hindered their practical application. Herein, inspired by the ultrafluidic and K^+^ ion-sieving flux through enzyme-gated potassium channels (KcsA) in biological plasma membranes, a metal-organic-framework (MOF-5) grafted with –ClO_4_ groups (MOF-ClO_4_) as functional enzymes is fabricated to mimic the ultrafluidic lipid-bilayer structure for gating Zn^2+^ ‘on’ and anions ‘off’ states. The MOF-ClO_4_ achieved perfect Zn^2+^/SO_4_^2−^ selectivity (∼10), enhanced Zn^2+^ transfer number (${{t}_{{\mathrm{Z}}{{{\mathrm{n}}}^{2 + }}}} = 0.88$) and the ultrafluidic Zn^2+^ flux (1.9 × 10^−3^ vs. 1.67 mmol m^−2^ s^−1^ for KcsA). The symmetric cells based on MOF-ClO_4_ achieve a lifespan of over 5400 h at 10 mA cm^−2^/20 mAh cm^−2^. Specifically, the performance of the PMCl-Zn//V_2_O_5_ pouch cell keeps 81% capacity after 2000 cycles at 1 A g^−1^. The regulated ion transport, by learning from a biological plasma membrane, opens a new avenue towards ultralong lifespan aqueous batteries.

## Teaser

A plasma membrane-inspired top layer is fabricated by grafting strong electronegative gating groups in metal-organic-frameworks for effective Zn^2+^ ion-sieving and ultrafluidic transport to regulate the dendrite-free Zn layer deposition for ultralong lifespan aqueous Zn-ion-batteries.

## INTRODUCTION

Aqueous Zn-ion-batteries (AZIBs) have been regarded as the alternative energy storage devices to Li-ion batteries, because of abundant Zn reserves, considerable capacity (820 mAh g^−1^), and low redox potential (−0.76 V vs. standard hydrogen electrodes) [1–4]. Unfortunately, the undesirable Zn dendrite growth and spontaneous side reactions, i.e. corrosion and hydrogen evolution reaction (HER), limit the large-scale application of AZIBs [5–7]. Specifically, when inorganic salts, such as ZnSO_4_, are used as the electrolyte, the fragile by-production Zn_4_SO_4_(OH)_6_·*x*H_2_O could further compromise battery performance [8]. These parasitic reactions cause irregular electric field distributions, lead to the growth of protrusions via a ‘tip-effect’, and finally reduce the battery life [9,10]. Recently, several metal-organic-framework (MOF)-related works have been reported for improving ZIBs performance, such as surface coating [11,12], electrolyte additives [13–15], substrate modifications [16,17], and separator design [18–20]. However, the realization of ultrafluidic ionic gates that control the flux of anions (gate off) and Zn^2+^ ions (gate on) should be a more promising aspect to regulate the ion transfer behaviors in order to reach a longer lifespan of the active-metal-based batteries [21,22]. Furthermore, the Zn deposition along the (002) crystal plane rather than the vertical (101) and (110) crystal planes can be achieved so as to avoid the vertical growth of dendrites [23,24]. The dendrite formation is widely accepted as a dynamic control process, it has been reported that fast Zn^2+^ flux and sluggish anion mobility would lead to a longer ‘sands time’, thus inhibiting dendrite growth [25,26], informing the importance of the recognizable Zn^2+^ ion-sieving ultrafluidity in suppressing dendrite growth in AZIBs.

In this work, we conceptually proposed a Zn^2+^ ion-sieving ultrafluidic method by applying a bioinspired membrane coating, inspired by ultrafluidic K^+^ flux through the biological plasma membrane potassium (KcsA) channels. Bioinspired design for functional materials and interface has been applied to study energy devices [27–31]. In well-evolved biological cells, the plasma membrane, composed of a thin layer of lipids and proteins surrounding the cell, has the capacity for selective ion penetration, such as KcsA channel, at a directional ultrafast K^+^ flux of 10^7^ ions s^−1^ (equivalent to 1.67 × 10^−3^ mol m^−2^ s^−1^) and an ion selectivity (K^+^/Na^+^) >1000 [29,32]. It is found that the ion superfluidity and ion-sieving function of the plasma membrane are realized by the enzyme-gated ion channels in sizes ∼0.35–1.5 nm [33]. Inspired by the ion-sieving KcsA channels, bioinspired membranes that enable Zn^2+^-sieving ultrafluidity but with anions-screening, were designed based on a MOF-5 with open channels at a size ∼6.7 Å [34]. However, the pristine MOF-5 has no ion-selective ability, due to the lack of a specific ‘enzyme-gate’. Herein, strongly electronegative –ClO_4_ groups are grafted onto the channels of MOF-5 (MOF-ClO_4_) as the ‘enzyme-gate’ via a solvent-assisted linker exchange (SALE) method [35]. The strong electronegativity of −ClO_4_ groups control Zn^2+^ ‘gate on’ to pass through the channels while gating off the anions via electrostatic repulsion. In the application, MOF-ClO_4_ nanoparticles were mixed with hydrophobic polystyrene (PS) and then fabricated into a thin membrane (PMCl) onto the Zn-electrode via electrospinning.

The bioinspired membrane achieved a Zn^2+^ flux of 1.9 × 10^−3^ mmol m^−2^ s^−1^, and a Zn^2+^/SO_4_^2−^ selectivity of 10. Due to the near-perfect Zn^2+^ ion-sieving and ultrafluidic properties, the bioinspired membrane guides the plating of the preferred Zn (002) facets. The symmetric cells displayed an ultra-long cycle life of over 5400 h at 10 mA cm^−2^/20 mAh cm^−2^. Remarkably, when assembled with a commercial V_2_O_5_ cathode into full cells, the coin full cell in a configuration of PMCl-Zn//V_2_O_5_ maintained 87% capacity (228 mAh g^−1^) after 2500 cycles at 2 A g^−1^ while the pouch cell reached 315 mAh g^−1^ at 1 A g^−1^ and retained 81% capacity after 2000 cycles even under bending, bending recovery, and damaging. Therefore, by learning from the ion-sieving functionality of the biological KcsA channel, an effective bioinspired electrode was designed to suppress the dendrite growth and maintain long-term stability resulting from Zn^2+^ ion-sieving ultrafluidity and preferential Zn (002) plane deposition. This bioinspired design provides an example of how to apply natural principles to designing artificial materials and devices. It sheds light on driving sustainable aqueous non-toxic metal batteries into real-world applications with greatly-enhanced lifespans.

## RESULTS AND DISCUSSION

### Fabrication of the bioinspired membrane

**Figure 1.**
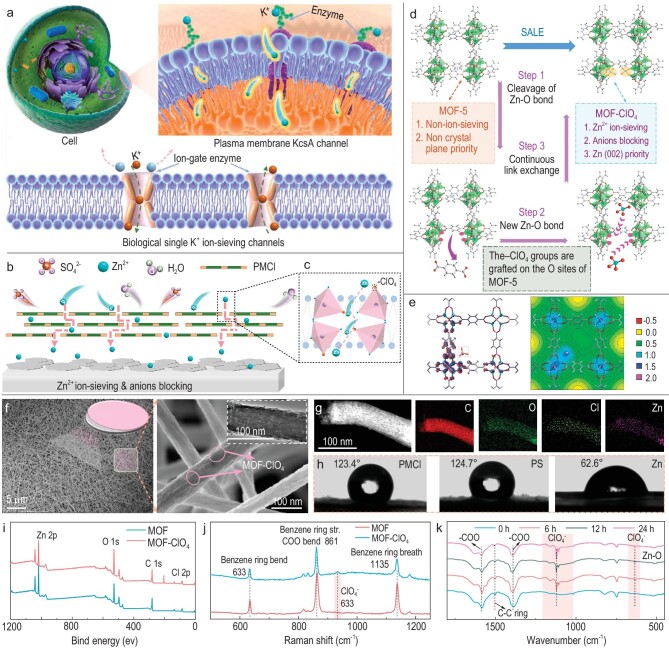
Bioinspired design of dendrite-free Zn-anode. (a) Schematic illustration of ultrafluidic transport of specific K^+^ ions through the enzyme-gated plasma membrane KcsA channel while screening undesired ions in biological cells; (b) bioinspired membrane with defined ion channels for Zn^2+^ ion-sieving and ultrafluidic transport; (c) ultrafluidic Zn^2+^ hopping path along the bioinspired –ClO_4_ group-gated channels; (d) synthesis of bioinspired materials by using MOF-5 via a solvent-assisted linker exchange (SALE) process, by which the –ClO_4_ groups are grafted onto the O-sites of the MOF-5 channels; (e) electron density difference and electrostatic potential mapping of the grafted MOF-ClO_4_; (f) scanning electron microscopy (SEM) image and high-resolution SEM image of PMCl fibers on the Zn electrode by electrospinning, and the inset is the TEM image of PMCl; (g) TEM and the corresponding elemental mapping of a PMCl fiber; (h) contact angle measurements of PMCl, PS and bare Zn surfaces; (i) XPS spectra collected on MOF-5 and MOF-ClO_4_ membranes and Cl 2p chemical states; (j) Raman spectra collected on MOF-5 and MOF-ClO_4_ membranes; (k) FT-IR spectra collected on MOF-ClO_4_ membranes with different SALE time periods.

Ion-gating behaviors have been widely identified in biological cells, in which a set of ultrafluidic ion channels with ion-sieving enzyme gates exist in the plasma membranes to regulate the ions in and out of the cells. As shown in Fig. 1a, the key component to realize the unique selectivity is the enzyme-gate formed by charged amino acids or proteins of the ion channels, which control the ‘gate on’ and ‘gate off’ states based on the charge, chemical properties, or size. Being inspired by the KcsA channel, a functional PMCl coating onto the Zn electrode with biomimicking ion-sieving was designed based on MOF-5, for regulating Zn^2+^ (002) facet nucleation, repelling the anions and H_2_O molecules caused by side-reactions (Fig. 1b and c). To realize the Zn^2+^-sieving, strongly electronegative −ClO_4_ groups (Fig. S2) were grafted onto the MOF-5 channels to mimic the enzyme-gate of the KcsA channels via the SALE process (Fig. 1d and Fig. S3). Figure 1e illustrates the calculated electron density difference of the MOF-ClO_4_ and the electrostatic potential (ESP) mapping, where the strong interaction between the link-site and the –ClO_4_ group is shown.

In analogy with the hydrophobic tail of the lipid-bilayer, hydrophobic PS was employed to glue the MOF-ClO_4_ nanoparticles (PMCl) (Fig. S4) by electrospinning, and the Zn anode was covered by interweaving PMCl nanofibers (PMCl-Zn) with less than ∼1 μm thickness (Fig. 1f and Fig. S5). The PMCl shows a rougher fiber surface than the bare PS fiber caused by the embedded MOF-ClO_4_ particles (Fig. S6). The high-resolution transmission electron microscopy (TEM) dark field image and corresponding elemental mapping (Fig. 1g) confirmed the homogeneous MOF-ClO_4_ embedment. Contact angle tests showed the hydrophobic surface of PMCl (123.4°), and PS (124.7°) compared with the bare Zn surface (62.6°) (Fig. 1h). Therefore, water could not penetrate the PMCl membrane (Fig. S7). As the X-ray photoelectron spectroscopy (XPS) shows, a Cl (2p) spectrum exhibits –ClO_4_ signals in MOF-ClO_4_ (Fig. 1i). Raman spectra were used to characterize the structural features of the pristine MOF-5 and the MOF-ClO_4_ (Fig. 1j), and the characteristic vibrational modes at 633, 861 and 1135 cm^−1^ were identified in both materials, further confirming the integrity of the framework after structural modification [36], which also coincided with the unchanged crystal structure of the MOF-ClO_4_ examined by X-ray diffraction (XRD) technique (Fig. S8). In the PMCl, however, a new mode found at 934 cm^−1^ standing for the *v*_1_ symmetric vibrational stretch of –ClO_4_ appeared. Fourier transform infrared (FT-IR) spectra were collected for MOF-ClO_4_ with different SALE times, where two extra peaks at 635 and 1100 cm^−1^ raised by the symmetric vibration of the –ClO_4_ linked with the O site of MOF-5 were identified (Fig. 1k).

### Ion-serving and ultrafluidity of bioinspired membranes

**Figure 2.**
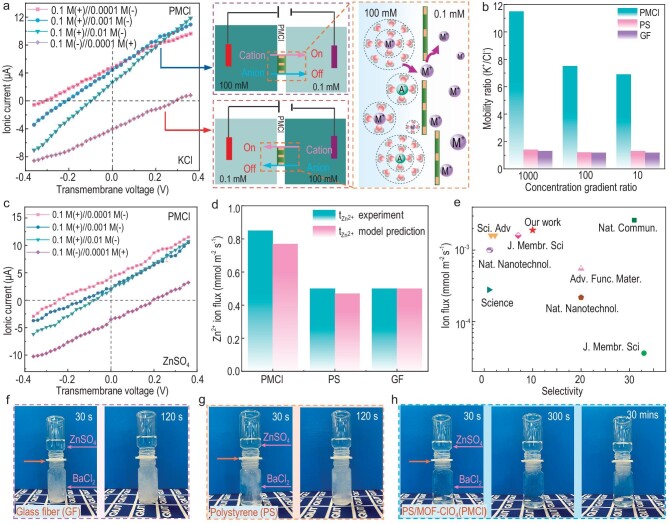
Ion-sieving property of bioinspired PMCl-Zn electrode. (a) Drift-diffusion *I–V* curves of PMCl membrane measured at various KCl concentration gradients, and illustrations demonstrating the ‘gate on’ and ‘gate off’ ionic behaviors; (b) mobility ratio, μ^+^/μ^−^, of bioinspired PMCl membrane and referential polystyrene (PS) and glass fiber (GF) membranes under concentration gradients of Δ = 10, 100 and 1000; (c) drift-diffusion *I–V* curves of PMCl membrane measured at various ZnSO_4_ concentration gradients; (d) Zn^2+^ ion flux of the three membranes determined experimentally (blue) and via the mathematical model (red); (e) comparing the Zn^2+^ ion flux and the selectivity (Zn^2+^/SO_4_^2−^) with other reported works (Table S3); (f) precipitation of GF membrane; (g) precipitation of PS; (h) precipitation of PMCl membranes. In (f–h), the solution in the top chamber is ZnSO_4_ solution (2 mL, all ions are under same gravity) and the bottom is BaCl_2_ solution, which is separated by the selected membranes as indicated.

The ion-gating performance of the bioinspired membrane was first tested by examining the current–voltage (*I–V*) characteristics in a two-chamber configuration with KCl electrolytes. In general, the ionic conductance of bulk electrolytes should be proportional to the concentration of electrolytes (Fig. S9). However, with the decreased KCl concentration (<0.1 M), the ionic conductance of the PMCl membrane significantly deviated from the bulk electrolyte, indicating that ionic conductance is governed by the surface charge. Next, the ion diffusion performance was further studied through a drift-diffusion experiment (Fig. 2a). Without an external voltage, significant net currents were generated associated with the flow of cations from high concentration to low concentration, affirming cation selectivity. At an ion concentration difference of 0.1 M vs. 0.0001 M, a net current of 4.78 μA was recorded (blue arrow), due to the effective ion-sieving property of PMCl membrane leading to a faster diffusion rate of the cations (Zn^2+^) to the anions (SO_4_^2−^). Switching the concentration gradient in an opposite direction, a net current of −4.14 μA was observed (red arrow). However, the net currents from the PS and GF membranes (Fig. S10) were a magnitude smaller, proving the cations-sieving and anions-screening resulted from the negatively charged PMCl membrane (schematic diagrams, Fig. 2a).

To quantitatively calculate the ion selectivity, the zero-current potential *E_m_* is used to define the mobility ratio of cations to anions (μ^+^/μ^−^) using the Henderson equation [37] (Table S1),


(1) \begin{eqnarray*}
\frac{{{{\mu }^ + }}}{{{{\mu }^ - }}} = - \frac{{{{z}_ + }}}{{{{z}_ - }}}\frac{{\ln\! \left( \Delta \right) - \frac{{{{z}_{F{{E}_m}}}}}{{RT}}}}{{\ln\! \left( \Delta \right) - \frac{{{{z}_ + }F{{E}_m}}}{{RT}}}},
\end{eqnarray*}

where *z*_+_ and *z*_−_ are the valences of cations and anions; *F* is the Faraday constant; *R* is the universal gas constant; *T* is the temperature; $\Delta $ is the ratio of concentration in the feed and permeate reservoirs. Figure 2b plots the μ^+^/μ^−^ values of PMCl, PS, and glass fiber (GF) membranes, and the values of the PMCl membrane were 11.5 (Δ = 1000), 7.5 (Δ = 100), and 6.9 (Δ = 10), respectively, which are 5–8 times higher than those of PS and GF membranes (SI appendix, Table S1). Furthermore, the cation selectivity of the PMCl membrane can be described by the cation transference number, *t_+_*. There is no cation selectivity when *t_+_* = 0.5 [29]. The *t_+_* value for the PMCl membrane was 0.96 (Δ = 1000), which is remarkably close to a perfect cation selection and far higher than that of the PS (0.59) and GF (0.57) membranes (Fig. S11). The higher *t_+_* for PMCl also indicates that an ion-hopping mechanism occurs for the ultrafluidic transport of the cations through the membrane. Figure S12 illustrates the current–voltage (*I–V*) curves at 0.01 M. The PS and GF membranes almost presented a linear *I–V* response, but the PMCl membrane showed a nonlinear *I–V* characteristic with a higher ionic rectification ratio of 1.7, compared with PS (0.78) and GF (0.79) membranes. In general, an ionic rectification ratio larger than 1.0 means a preferential cation transport [38]. Therefore, according to the quantitative ion transport characterizations, we confirm that the bioinspired PMCl membrane has a perfect cation-sieving ability.

Figure 2c shows the drift-diffusion *I–V* curves as the variation of ZnSO_4_ concentration differences, where the Zn^2+^ ion-sieving shows a similar trend as that of K^+^ with a ${{{\mathrm{\mu }}}^{{\mathrm{Z}}{{{\mathrm{n}}}^{2 + }}}}/{{{\mathrm{\mu }}}^{{\mathrm{SO}}_4^{\ 2 - }}}$of 11.3 (Δ = 1000), proving a Zn^2+^ ‘gate on’ and SO_4_^2−^ ‘gate off’ result. The Zn^2+^ ion-sieving effect of the PMCl membrane was further verified by ion permeation resistance in an H-shape device filled with deionized water and 1 M ZnSO_4_ in each chamber and separated by the membranes (Fig. S13). The ion concentration was examined by the inductively coupled plasma (ICP) and ion chromatography (IC) techniques. Based on the membrane properties (Table S2) and the concentration of Zn^2+^ and SO_4_^2−^ passing through the membrane with time (Fig. S14), the average Zn^2+^ flux of the PMCl membrane was 1.9 × 10^−3^ mmol m^−2^ s^−1^ and Zn^2+^/SO_4_^2−^ selectivity was 10, which matches well with a mathematical model applied for predicting the ion flux and Zn^2+^ transfer membranes (${{t}_{{\mathrm{Z}}{{{\mathrm{n}}}^{2 + }}}}$) of micro-heterogeneous membranes (SI) [39] (Fig. 2d). For the PS and GF membranes, the Zn^2+^ flux was 4.1 × 10^−4^ and 4.2 × 10^−4^ mmol m^−2^ s^−1^, respectively, an order of magnitude lower than the PMCl (Fig. S15); besides, there was no ion selectivity performance. Based on this test, the experimental ${{t}_{{\mathrm{Z}}{{{\mathrm{n}}}^{2 + }}}}$ were 0.82 (PMCl), 0.5 (PS), and 0.49 (GF), respectively (Fig. S16). Surprisingly, the ion flux and ion selectivity ability of the PMCl membrane are comparable to some of the best-performing ion-sieving membranes (Fig. 2e, Table S3).

To supply a more distinct comparison, precipitation tests were conducted on different membranes to detect the SO_4_^2−^ rejection properties. The 1 M ZnSO_4_ solution (3 mL) in the top vessel was separated from the saturated BaCl_2_ solution in the bottom vessel by the GF (Fig. 2f), PS (Fig. 2g), and PMCl (Fig. 2h) membranes. After only 30 s, precipitates formed in the bottle vessels separated by both GF and PS membranes, resulting from the reaction between the Ba^2+^ and the permeated SO_4_^2−^. For the PMCl-separated vessels, no significant precipitate formed even up to 30 min (Fig. 2h). As the SO_4_^2−^ ions in the three vessels had the same gravity, it could only be attributed to the repulsion effect that controls the passage of SO_4_^2−^ ions through the PMCl membrane. To reveal the side reactions on the Zn surface, PMCl membrane-covered Zn electrode (PMCl-Zn), PS-covered Zn electrode (PS-Zn), and bare Zn were soaked in 1 M ZnSO_4_ electrolyte for 7 days. Figure S17 revealed the Zn surface morphologies—the PMCl-Zn electrode remained smooth, but the PS-Zn and the bare Zn electrodes were covered with by-products. The results indicate that the PMCl membrane could prevent the anions and H_2_O molecules from generating side reactions.

### Regulated Zn (002) dendrite-free deposition on bioinspired electrodes

The electrodes were assembled into symmetrical cells to examine their electrochemical properties. The electrical resistivity (ρ) of the PMCl membrane measured via a two-electrode configuration was about 7.7 × 10^4^ Ω cm (∼1.3 × 10^−5^ S cm^−2^, Fig. S18), nearly an electron insulator. From the electrochemical impedance spectroscopy (EIS) tests of blocking cells, the PMCl membrane showed good ion conductivity (∼1.2 × 10^−3^ S cm^−2^, Fig. S19). The ${{t}_{Z{{n}^{2 + }}}}$ in the AZIBs was also calculated (Fig. S20). Lower ${{t}_{Z{{n}^{2 + }}}}$ values were observed for the bare Zn (0.64) and the PS-Zn electrodes (0.69). For the PMCl-Zn electrode, the ${{t}_{Z{{n}^{2 + }}}}$ value improved to 0.88, which is a bit lower than the cation transfer number measured from the KCl solution, yet an incredibly significant number.

**Figure 3.**
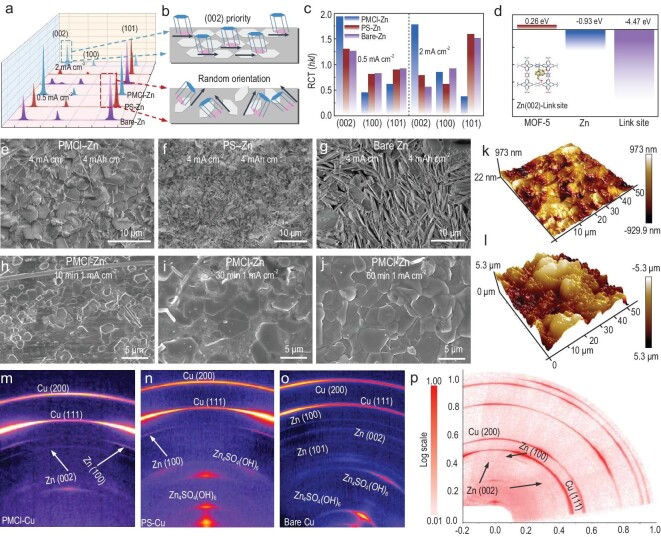
Crystal plane and surface topography characterizations on the deposition of Zn. (a) XRD patterns of electrodes cycled at 0.5 and 2 mA cm^−2^; (b) schematic illustration of preferred orientations of Zn deposition on different substrates; (c) calculated RTC values from the XRD peak intensity; (d) Zn (002) plane adsorption energy at different sites; (e) morphology of cycled PMCl-Zn electrode; (f) morphology of cycled PS-Zn electrode; (g) morphology of cycled bare Zn electrode, after 100 cycles at 4 mA cm^−2^/4 mAh cm^−2^; (h) morphology of PMCl-Zn anode cycled at 1 mA cm^−2^ for 10 min; (i) PMCl-Zn anode cycled for 30 min; (j) PMCl-Zn anode cycled for 60 min; (k) AFM mapping on the deposited Zn surface on PMCl-Zn; (l) AFM mapping of bare Zn, after 200 cycles plating/stripping (1 mAh cm^−2^, 1 mA cm^−2^); 2D WAXS patterns of (m) PMCl-Zn anode, (n) PS-Zn anode and (o) bare Zn anode, after first cycling in asymmetrical cells at 2 mAh cm^−2^/0.5 mA cm^−2^; (p) WR-RSM mapping showing Zn (002) texture along the Sz direction on the sample normal direction.

The crystal plane orientation and morphology of Zn plating were examined by symmetrical cells. Figure 3a displays the XRD patterns of the three electrodes after cycling at 0.5 and 2 mA cm^−2^, respectively. Three major diffraction peaks at 36.5°, 39.2°, and 43.4° correspond to the (002), (100), and (101) diffractions of the plated Zn layers [40]. Zn (002) crystal plane is almost paralleled to the substrate (Fig. 3b). The relative texture coefficients (RTC) of the plated Zn are calculated as the following equation [41]:


(2) \begin{eqnarray*}
RT{{C}_{\left( {hkl} \right)}} = \frac{{{{I}_{\left( {hkl} \right)}}/{{I}_{0\left( {hkl} \right)}}}}{{\frac{1}{n}\sum \left( {{{I}_{\left( {hkl} \right)}}/{{I}_{0\left( {hkl} \right)}}} \right)}},
\end{eqnarray*}

where *I_(__hkl__)_* is the diffraction intensity of the textured samples and *I*_0_*_(__hkl__)_* is the diffraction intensity of the randomly oriented sample (Table S4). The RTC values for PMCl-Zn increased from 1.75 (2 mA cm^−2^) to 1.93 (0.5 mA cm^−2^), and a preferred Zn (002) orientation is more obvious than the other two electrodes (Fig. 3c and Fig. S21). The DFT calculation demonstrates that the Zn (002) texture on the PMCl-Zn electrode is as a result of the much more favorable binding energy of Zn (002) cluster with the –ClO_4_ modified linker sites (−4.47 eV) than the bare Zn (−0.93 eV) and the unmodified MOF-5 (0.26 eV) (Fig. 3d and Fig. S22).

The deposited Zn morphology was verified by SEM (Fig. 3e–g). The horizontal Zn plates were observed for the PMCl-Zn anode at 4 mA cm^−2^/4 mAh cm^−2^ (Fig. 3e). For the PS-Zn (Fig. 3f) and bare Zn (Fig. 3g and Fig. S23) anodes, substantial amounts of vertically aligned Zn-dendrites were formed. The Zn plating progress on the PMCl-Zn anode was revealed by cycling at 1 mA cm^−2^ with time (Fig. 3h–j). Small-sized hexagonal Zn (002) crystals were exposed and then grew larger and stacked up into a thicker layer beneath the PMCl membrane (Fig. S24). Remarkably, as evidenced by the atomic force microscopy (AFM) 3D height mapping, a smooth surface constructed by the horizontally plated Zn crystals was examined for PMCl-Zn (Fig. 3k), but a very rough surface formed on the bare Zn substrate (Fig. 3l and Fig. S25).

The Cu substrate was employed to prevent the deposited Zn diffraction signal from interfering with the Zn substrate. Similarly, by applying asymmetrical cells, the Cu substrate covered with PMCl membrane (PMCl-Cu) exhibited Zn (002) deposition, while dendritic Zn layers were formed on the PS-Cu and bare Cu electrodes (Fig. S26). Furthermore, 2D wide-angle X-ray scattering (WAXS) and wide-range reciprocal space mapping (WR-RSM) combined from 2D scans at different sample tilting angles in the Eulerian Cradle were performed. The WAXS patterns of the deposited Zn on PMCl-Cu (Fig. 3m) displayed discontinuous Zn (002) diffractions in the plane ring, indicating a Zn (002) texture [42]. On the PS-Cu and bare Cu anodes, however, the (002) diffraction ring intensities are continuous (Fig. 3n and o), and strong diffraction rings are for Zn_4_SO_4_(OH)_6_. This means that voluminous anions and H_2_O molecules contact the Zn surface and form Zn_4_SO_4_(OH)_6_. As the WR-RSM shows (Fig. 3p), the Zn (002) reflection only appeared in the sample's normal direction. It is the PMCl membrane instead of the substrate that induced Zn (002) plane exposure.

### Battery performance of the bioinspired electrode

**Figure 4.**
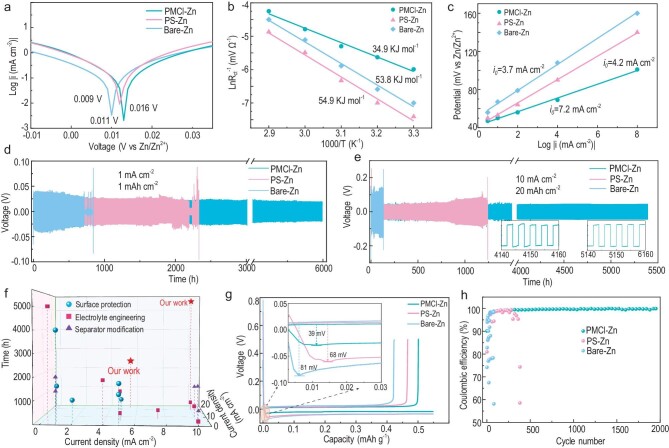
Electrochemical performance of symmetric cells and half-cells with bioinspired PMCl-Zn, PS-Zn and bare Zn electrodes. (a) Polarization curves of the PMCl-Zn, PS-Zn and bare Zn electrodes in two-electrode cells; (b) calculated desolvation activation energies by the Arrhenius equation; (c) plot of potentials vs. current density for calculating the exchange current density *i* = 2*i*_0_η*F*/*RT*, where *η* is the total overpotential, *i* is the running current density and *i*_0_ is the exchange current density; (d) cycling performance of the symmetric cells examined at 1 mA cm^−2^ and 1 mAh cm^−2^; (e) cycling performance at 10 mA cm^−2^ and 20 mAh cm^−2^; (f) performance comparison of the bioinspired electrode with other works (Table S5); (g) magnified view of the 1st cycle; (h) coulombic efficiencies for Cu//Zn, Cu//PS-Zn,and Cu//PMCl-Zn electrodes at 0.5 mA cm^−2^.

The HER and corrosion were further investigated by a linear polarization experiment (vs. Zn/Zn^2+^) by symmetrical cells (Fig. 4a). The corrosion potential of the PMCl-Zn electrode was 0.016 V, which is higher than that of the PS-Zn (0.011 V) and the bare Zn (0.009 V) electrodes. A higher corrosion potential means a lower tendency towards corrosion reactions. After 50 cycles, no formation of Zn_4_SO_4_(OH)_6_•*x*H_2_O on the PMCl-Zn electrode is detected by XRD, different from the situations of PS-Zn and the bare Zn (Fig. S27), which indicates that the corrosion reaction is disrupted by the PMCl membrane. To quantitatively inspect the HER reaction, *in-situ* gas chromatography (GC) analysis was applied to dynamically detect the H_2_ evolution during the charging/discharging process, and as the contour map shows (Fig. S28), the HER was principally suppressed by the PMCl-Zn when compared with bare Zn.

The Zn deposition kinetics was depicted by the activation energy (*E*_a_), according to the EIS with temperature tests (Fig. S29) [43]. Using Arrhenius law, an *E_a_* value of 34.9 kJ mol^−1^ for the PMCl-Zn electrode was estimated (Fig. 4b), lower than that of the PS-Zn (54.9 kJ mol^−1^) and the bare Zn anodes (53.8 kJ mol^−1^). The exchange current density (*i_0_*) is another critical kinetic factor [44]. The higher *i*_0_ value indicates more rapid redox reaction. As shown in Fig. 4c, the PMCl-Zn electrode had a higher *i*_0_ value (7.2 mA cm^−2^) than the PS-Zn (4.2 mA cm^−2^) and the bare Zn anode (3.7 mA cm^−2^), showing that the bioinspired PMCl membrane significantly facilitates the transport of Zn^2+^.

Before evaluating the cycling stability of the symmetrical cells, the rate performance was examined at current densities varied over 0.5–20 mA cm^−2^ (Fig. S30). Benefiting from the ion-sieving ultrafluidity, the PMCl-Zn cell always exhibited the lowest voltage hysteresis. Figure 4d compares the cycling performance at 1 mA cm^−2^/1 mAh cm^−2^, the bare Zn and PS-Zn electrodes presented dramatic voltage oscillations and lost function within 1000 h and 2000 h, respectively. However, the PMCl-Zn electrode delivered excellent stability up to 6000 h. Even at 10 mA cm^−2^ (20 mAh cm^−2^) (Fig. 4e), the PMCl-Zn electrode maintained voltage hysteresis of only ±0.05 V for 5400 h without obvious impedance change. It shows that the PMCl membrane was not damaged, and horizontal Zn deposition was observed (Fig. S31), in contrast to the PS-Zn (1250 h, ±0.1 V) and the bare Zn (200 h, ±0.15 V). Under a longer deposition time of 4 h at 5.5 mA cm^−2^ (Fig. S32), the PMCl-Zn electrode also maintained 2000 h and small (±0.04 V) voltage hysteresis. No morphology changes of PMCl fibers and no falling off of the MOF particles were identified (Fig. S33). The comprehensive performance of the PMCl-Zn electrode surpasses most of the recently reported AZIBs improved using various other strategies (Fig. 4f, Table S5).

The coulombic efficiency (CE) for Zn plating/stripping was revealed by asymmetrical cells. The Cu//PMCl-Zn half-cell achieved a cycling life of over 3800 h at 0.5 mA cm^−2^/0.5 mAh cm^−2^ (Fig. S34). In contrast, the Cu//PS-Zn cell could not maintain a stable supply after 800 h, and <200 h for the Cu//Zn cell. Figure 4g illustrates the magnified curves of the first cycle. The overpotential of the Cu//PMCl-Zn was 39 mV, less than the value of Cu//PS-Zn (68 mV) and Cu//Zn (81 mV) cells. In addition, the overpotential values for the 10th–2000th hours were almost the same as the first cycle, revealing the excellent reversibility of the PMCl-Zn anode (Fig. S35). The CE of the Cu//Zn decayed rapidly to 58% after 50 cycles and the one with PS-Zn anode dropped to 60% within 450 cycles. In contrast, an average CE of 99.5% after 1800 cycles could still be maintained for the Cu//PMCl-Zn cell (Fig. 4h). Figure S36 shows the nucleation overpotential, where the PMCl-Zn anode delivered the lowest value, i.e. 15 mV at 0.5 mA cm^−2^. A lower nucleation overpotential means more uniform Zn^2+^ deposition.

**Figure 5.**
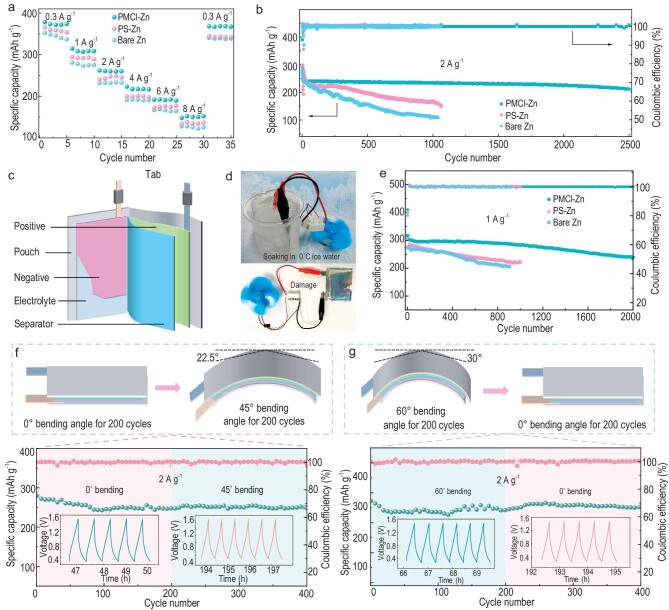
Electrochemical performance of PMCl-Zn//V_2_O_5_ full cells. (a) Rate performance of a full coin battery at current densities from 0.3 to 8 A g^−1^; (b) cycling performance of a PMCl-Zn//V_2_O_5_ coin cell at 2 A g^−1^; (c) structure diagram of a 5 cm × 3 cm pouch-type cell; (d) powering up a mini-fan using a pouch cell while soaking in ice water and in damaged state, respectively; (e) cycling performance of the pouch cell at 1 A g^−1^. The carton and cycling performance of the flexible battery under different bending angles: (f) 0° bending for 200 cycles and then 45° bending for 200 cycles; (g) 60° bending for 200 cycles and then 0° bending for 200 cycles. The insets show the specific cycles.

To demonstrate the feasibility of the PMCl-Zn anode in practical applications, we evaluated the performance of the full batteries in both coin cells and pouch cells with commercial V_2_O_5_ as the cathode. Figure 5a shows the rate performance over 0.3–8 A g^−1^ of the coin cells in configurations of PMCl-Zn//V_2_O_5_, PS-Zn//V_2_O_5_, and Zn//V_2_O_5_. The full cells with PMCl-Zn, PS-Zn, and bare Zn anodes delivered a capacity of 312.2, 276.4, and 276.1 mAh g^−1^, respectively, at 1 A g^−1^. For the PMCl-Zn//V_2_O_5_ cell, the capacity of 152.5 mAh g^−1^ could still be achieved at 8 A g^−1^. Figure 5b presents the cycling stability of coin cells. While both the coin cells with PS-Zn and Zn anodes lost more than 50% of their capacity merely over 1000 cycles, the coin cells with PMCl-Zn anode enabled a stable cycling performance with 87% capacity retention (228 mAh g^−1^) after 2500 cycles at 2 A g^−1^.

Flexible pouch cells make them ideal for use in portable electronic devices and electric vehicles with their compact and lightweight design. We assembled the aqueous Zn-ion full batteries into pouch cells, in the configuration shown in Fig. 5c, to further verify the feasibility of the PMCl-Zn for wider application scenarios. It is interesting that the pouch cells with a PMCl-Zn anode and a V_2_O_5_ cathode successfully powered a mini-fan under extremely harsh conditions, for example, soaked in 0°C ice water, at which the aqueous 2 M ZnSO_4_ electrolyte still maintained at a liquid state, and a damaged state (Fig. 5d). Surprisingly, the cycling performance of the pouch cell (N/P ∼10) at a V_2_O_5_ mass loading of ∼8.7 mg cm^−2^ reached 315 mAh g^−1^ at 1 A g^−1^ and maintained 81% capacity retention and over 99.5% CE after 2000 cycles. The pouch cells without PMCl membranes, however, exhibited rapid capacity decay of more than 50% after 1000 cycles as those coin cells (Fig. 5e). Furthermore, Fig. S37 shows the Zn surface topography from the pouch batteries after 500 cycles, where the PMCl-Zn anode maintained a smooth surface with horizontal Zn (002) oriented plating (Fig. S37a), while dendritic surfaces formed on both the PS-Zn (Fig. S37b) and the bare Zn electrodes (Fig. S37c). The superior specific capacity and cycling performance of the pouch cell demonstrated the practical application potential of the designed bioinspired electrode. To further highlight the flexibility of the PMCl-Zn anode, the performance of the pouch cell was examined under different bending and recovery states. As shown in Fig. 5f and g and Fig. S38, the discharge capacity of the pouch cells remained nearly unchanged after bending at 45°, recovering from 60°, and twisting, indicating the outstanding mechanical stability and flexibility of the pouch cells with bioinspired electrodes.

## CONCLUSION

In summary, inspired by the ion-sieving and ultrafluidity of the biological KcsA channels, a bioinspired PMCl membrane was designed for dendrite-free AZIBs. With the bioinspired Zn^2+^ ion-sieving ultrafluidic properties, the PMCl membrane achieved a Zn^2+^ flux of 1.9 × 10^−3^ mmol m^−2^ s^−1^ and a Zn^2+^/SO_4_^2−^ selectivity of 10. Besides, the bioinspired membranes successfully modulated the Zn^2+^ deposition in a (002) preferred orientation and inhibited corrosion and HER. Benefiting from these advantages, the bioinspired PMCl-Zn electrode delivered an ultralong lifespan of over 5400 h at 10 mA cm^−2^/20 mAh cm^−2^, and an average CE of over 99.5%. The coin full cell in a configuration of PMCl-Zn//V_2_O_5_ maintained 87% capacity (228 mAh g^−1^) after 2500 cycles at 2 A g^−1^. When assembled into pouch cells for wider applications in potential portable devices and electric vehicles, the PMCl-Zn//V_2_O_5_ pouch cells reached a capacity of 315 mAh g^−1^ at 1 A g^−1^ with 81% capacity retention after 2000 cycles. It is worth noting that the pouch cells provide a stable electric supply in both a harsh environment and a damaged state, demonstrating the safety and practical application potential of this sort of bioinspired battery. This bioinspired design not only provides an example of how to apply natural principles to designing artificial materials and devices but also sheds light on driving sustainable aqueous non-toxic metal batteries into real-world applications with a much enhanced lifespan.

## METHODS

The detailed preparation and characterization methods of materials are available in the online Supplementary file.

## Supplementary Material

nwae199_Supplemental_File
