# Supplementary material for: Zn-ion ultrafluidity via bioinspired ion channel for ultralong lifespan Zn-ion battery
Source: Natl Sci Rev. 2024 Jun 12;11(8):nwae199. doi: 10.1093/nsr/nwae199 (PMC11267990; doi:10.1093/nsr/nwae199)
Supplement: nwae199_Supplemental_File [file nwae199_supplemental_file.docx]

Supporting Information

**Zn-ion ultrafluidity via bioinspired ion channel for ultralong lifespan Zn-ion battery**

Fan Zhang ^a^, Ting Liao ^e,^ *, Dongchen Qi ^a, c^, Tony Wang ^b, c^, Yanan Xu ^b,c^, Wei Luo ^d^, Cheng Yan ^e^, Lei Jiang ^f, g *^, Ziqi Sun ^a,^ *

^a^ School of Chemistry and Physics, Queensland University of Technology, George Street, Brisbane, QLD 4000, Australia. E-mail: [ziqi.sun@qut.edu.au](mailto:ziqi.sun@qut.edu.au)

^b^ Central Analytical Research Facility, Queensland University of Technology, QLD 4000, Australia

^c^ Centre for Materials Science, Queensland University of Technology, 2 George Street, Brisbane 4000, Australia

^d^ State Key Laboratory for Modification of Chemical Fibers and Polymer Materials, College of Materials Science and Engineering, Donghua University, Shanghai, 201620, P. R. China

^e^ School of Mechanical Medical and Process Engineering, Queensland University of Technology, George Street, Brisbane, QLD 4000, Australia. E-mail: [t3.liao@qut.edu.au](mailto:t3.liao@qut.edu.au)

^f^ Key Laboratory of Bio-inspired Materials and Interfacial Science, Technical Institute of Physics and Chemistry, Chinese Academy of Sciences, Beijing, 100190 P. R. China

^g^ School of Future Technology, University of Chinese Academy of Sciences, Beijing, 100190 P. R. China. E-mail: [jianglei@mail.ipc.ac.cn](mailto:jianglei@mail.ipc.ac.cn)

**Experimental Procedures**

Preparation of MOF-5: MOF-5 was prepared according to the reported procedure with slight modification [1]. Zinc nitrate hexahydrate (4.50 g, 15.0 mmol) and 1,4-benzenedicarboxylic acid (0.83 g, 5.0 mmol) were dissolved in 490 mL of DMF and 10 mL H_2_O in a 1000 mL jar with a teflon lined lid. The reaction mixture was heated in an oven at 100 °C for 7 h to yield cube-shaped crystals. The solvent was decanted, and the remaining solid was washed three times with 500 mL of anhydrous DMF, each time letting the solid soak in DMF for 8 h. The DMF was then decanted, and the solid washed three times with 500 mL of CHCl_3_, again each time letting the solid soak in CHCl_3_ for 8 h. After the final CHCl_3_ wash, the solvent was decanted and the included CHCl_3_ was removed under vacuum to give colorless cube-shaped crystals.

Preparation of MOF-ClO_4_ by SALE: When the prepared Zn-MOF-5 powder was placed in the mixed Zn(ClO_4_)_2_ solution (V/% DMF : H_2_O = 4 : 1) for stirring, where the mass ratio of MOF-5 to Zn(ClO_4_)_2_ is 1: 10. And the reaction time was set as 6 h at the room temperature. Then the particles were washed by DMF to remove the extra Zn(ClO_4_)_2._ All of the products were collected by centrifugation at 3000 r.p.m for 6 mins and dried in a 60℃ oven.

Preparation of PMCl: PS (polystyrene, average molecular weight = 192000) precursor solution was prepared by dissolving PS in DMF, (PS/DMF=1:4). The mixture was stirred for 12 h. Then the MOF-ClO_4_ was added into the PS solution (mass ratio: PS:MOF-ClO_4_=1:1), keep stirring for another 6 h. Finally, the electrospinning was executed by a biomedical pump for 10, 20, 30, and 60 mins. (Voltage: 12 kV, flow rate: 0.8 mL/h, distance between syringe needle and collecting plate: 15 cm). The Zn (d=12 mm) or Cu foil was adhered on the aluminum foil surface to be covered by the fiber directly.

**Material Characterizations**

The crystalline structures and pole figures were examined by an X-ray diffractometer (XRD, Bruker D8 Advanced) with Cu Kα radiation. The Wide Range Reciprocal Space Mapping was collected on a Rigaku SmartLab diffractometer under CuKα radiation. The primary beam was conditioned by a CBO-PB 0.5mm pinhole followed by CBO-f poly-capillary. An 80mm snout was used as the final spot size control. The Zn coated on Cu foil was aligned in the center of a χ-φ sample cradle. A Hypix3000 pixel detector was used to collect Debye Curves from the sample in 2D mode at different χ tilts. The result 2D frames were χ-expanded in Rigaku 2DP and merged into WRRSM in Rigaku 3DE. The morphologies of the bare Zn foil and the bioinspired Zn electrodes were observed by a field emission scanning transmission electron microscope (SEM, TESCAN/MAIA3, Czech) and a transmission electron microscope (TEM, JEM-2100F JEOL, Japan) and Atomic force microscope (AFM, Bruker’s Dimension Icon). The Elemental analysis was performed on an X-ray photoelectron spectrometer (XPS, Thermo ESCALAB 250 Xi, Al Kα radiation, h*v* = 1486.6 eV, America). Contact angles were measured on a Dataphysics OCA15 wettability tester. The final solution in the permeate chamber was used to determine the transport rate by chemical analysis using inductively coupled plasma (ICP) (Profile Spec, Leeman, USA) for cations, ion chromatography (IC) (ICS-90, Dionex, USA) for anions. Gas chromatography (GC, Agilent 8890B) was conducted in-situ collecting the gas production during Zn plating/stripping process. The cyclic voltammetry (CV), linear scan voltammetry (LSV), chromoamperograms (CAs), and A.C. impedance measurements were carried out by utilizing a CHI 760e electrochemical workstation. The charge-discharge experiments were performed on a Land BT2000 battery test system at room-temperature.

For the full battery test, the electrodes were prepared by mixing commercial V_2_O_5_, acetylene black, and polyvinylidene fluoride (PVDF) at a weight ratio of 80%:10%:10%, the loading mass of V_2_O_5_ is about 2.85 mg cm^-2^. The mixture was dispersed in a small amount of methyl-2-pyrrolidinone (NMP) solvent by grinding in an agate mortar to achieve a stably homogeneous paste. The slurry was pasted on stainless steel foil and dried at 100 °C for 12 hours in a vacuum oven. The cells were galvanostatically charged/discharged over the voltage range of 0.3–1.5 V vs. Zn/Zn^2+^ at different current densities on Land CT2001A battery testers. The pouch-type batteries were assembled by sandwiching the electrolyte and the separator between the 3 cm × 5 cm V_2_O_5_ (loading mass: ~8.7 mg cm^-2^) cathode and the bioinspired PMCl-Zn (Zn thickness =100 µm) anode and sealed by Al-plastic films. The dosage of the electrolyte was about 1.5 mL.

**Ion Transport Properties Tests**

The cation selectivity was studied by testing the I–V properties on a picoammeter (Keithley 2450). During the testing, the separator was mounted between the two chambers of the electrochemical cell with concentration gradient KCl (ZnSO_4_) (0.0001M-1M) as the electrolytes. The KCl aqueous solution was chosen as the electrolyte for this test as the close values of diffusion coefficient between K^+^ (1.96 × 10^−9^ m^2^ s^−1^) and Cl^−^ (2.03 × 10^−9^ m^2^ s^−1^). Also, two Ag/AgCl electrodes were used to apply a transmembrane voltage. A bias voltage from −1 to +1 V was applied to gain insights into the ion transport properties. Before test, the membranes were dipped in DI water for 24 h due to the poor hydrophily.

Drift-diffusion experiments were conducted with the applied voltage from -0.35-0.35 V vs Ag/AgCl electrodes. The membranes were mounted between two chambers, one of which, facing the base, was filled with 0.1 M KCl solution, while the other was filled with 0.01, 0.001, and 0.0001 M KCl solution, respectively. From the I-V curves, the measured reverse potential (that is, zero-current potential, E_m_) was obtained (Figure S1, Table S1). During these measurements, a redox potential was generated at the electrodes because of the concentration gradient across the membrane; thus, real E_m_ is subtracted from the redox potential E_redox_ (real E_m_ = E_m_ − E_redox_), which is calculated as follows [2]:

$$E_{redox}=\frac{RT}{zF}ln\Delta\frac{\gamma_{H}C_{H}}{\gamma_{L}C_{H}}$$

where R, T, and F are the universal gas constant, temperature, and the Faraday constant, respectively. The $\gamma_{H}$ with subscript of and $\gamma_{L}$ is the activity coefficient of electrolyte solutions (KCl) at high concentration (C_H_) and low concentration (C_L_), respectively. The concentration gradient of the electrolyte:$\Delta$=*C_H_/C_L_*.


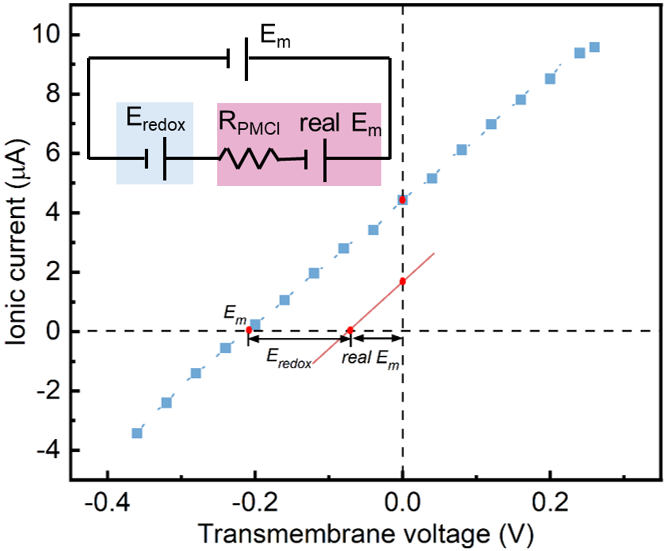


Figure S1. The drift-diffusion I-V curves under a 100-fold KCl gradient.

Ion transference number. The property of cations through the negatively charged PMCl could be described by the cation transference number, which is given as [2]:

$$t_{+}=\frac{1}{2}(\frac{E_{m}}{\frac{RT}{zF}ln\frac{\gamma_{H}C_{H}}{\gamma_{L}C_{L}}}+1)$$

where the E_m_ refers to the real value and all the parameters can be found in Table S1.

**The mathematical model** for phosphate ion transport was developed based on the microheterogeneous model for ion exchange membranes. The ion flux, *J_i_*, of each ion was calculated by the slope of concentration vs time during the penetration experiment (m, moles/s), normalized by the membrane surface area (*A_m_*) $J_{i}=$m/$A_{m}$,$mol m^{-2}s^{-1}$. Traditionally, the model describes two distinct phases within the membrane - a “gel phase” and an “intergel solution phase”. The gel phase encompasses the microporous regions containing the polymer chains with fixed charges, with the counter and co-ion containing solution balancing these fixed charges; the intergel phase consists of the meso- and macroporous spaces filled with electroneutral equilibrium solution (equivalent to the bulk external solution). The ion flux density of species i (counter/ co-ion), $J_{i}$. In each phase (more exactly, in each element of those phases with dimensions of 10-1000 nm) the Nernst-Planck equation written for one-dimensional diffusion is valid [3][4]:

$$J_{i}=-L_{i}^{*}\frac{{du}_{i}}{d_{x}}$$

Where, $L_{i}^{*}$ is the effective conductance coefficient characterizing the multiphase system (similar to a diffusion coefficient), μ_i_ is the electrochemical potential of species i, and x is the differential length under consideration in the direction of transport. Two cross-sections normal to axis x is drawn through the points with coordinates x and x + $d_{x}$ inside the system. The distance $d_{x}$ between cross-sections is chosen in such a way that it should be considerably larger than the dimensions of separate phase elements and, at the same time, be small enough so that the variations of conductance coefficients in the distance $d_{x}$ are negligible. The electrochemical potential, μ_i_, can be presented as a function of concentration of species i, *C_i_*, and electrical potential φ, as [5]:

$J_{i}=-L_{i}^{*}*(\frac{du_{i}^{0}}{dx}+RT\frac{dC_{i}}{dx}+Z_{i}F\frac{d_{\varphi}}{d_{x}}$)

Where, $u_{i}^{0}$ is the standard chemical potential, $Z_{i}$ is the charge of species *i*, and *F, R*, and *T* are the Faraday’s constant (C/mol), gas constant (J/mol/K), and temperature (K), respectively. Using the microheterogeneous model for the two-phase system where the superscript *g* refers to the ‘gel phase’ and the superscript ‘*int*’ refers to the intergel phase within the membrane, The gel phase is composed of charged groups (MOF-ClO_4_) and the polymer chains (PS) to which they are bound; the interstitial phase is the void between the elements of the gel phase. The source of the void could be interstices, pores, and structural defects. Likewise, the interstitial phase is assumed to be the space filled with electro-neutral solution when the membranes are hydrated with salt solutions and is responsible for the transport of co-ions through PMCl membrane. $L_{i}^{*}$ is calculated as:

$$L_{i}^{*}=[f_{g}(L_{i}^{g})^{\alpha}+f_{int}(L_{i}^{int})^{\alpha}]^{\frac{1}{\alpha}}$$

Where, α is the structural parameter of the membrane, which varies from -1 to 1 and

represents the connectivity of the two phases (where -1= series and 1= parallel), $f_{g}$ and $f_{int}$ are the volume fractions of the gel and intergel phases, respectively, which were approximated from the water uptake capacity of the membrane. Water uptake by the membranes was determined by noting the weight after soaking them in DI water for 48 hours (*w_w_*, g), followed by weighing the dried membranes (*w_d_*, g) after placing them in a vacuum oven at 50 ºC for 48 hours. The water uptake (*wu*, g water/g of dry polymer) was given by [6] [7]:

$$w_{u}=\frac{w_{w}-w_{d}}{w_{d}}$$

$$V_{water}=\frac{w_{u}*w_{d}}{\rho}$$

$$V_{gel}=\frac{w_{d}}{\rho}$$

$$f_{int}=\frac{V_{water}}{V_{water}+V_{gel}}$$

$$f_{g}=1-f_{int}$$

$$t_{+}^{*}=\frac{L_{+}^{*}}{L_{+}^{*}+L_{-}^{*}}$$

Where, Where, ρ is the density of pure water (g/L). $V_{water}$ is the volume of water taken up by the membrane during water uptake measurements assuming the water is present in the intergel region and $V_{gel}$ is the volume of the polymer gel phase. The value of $f_{int}$ and $f_{g}$ obtained using this approximation fall within the acceptable range reported in literature [8], [9]. $L_{i}^{g}$ and $L_{i}^{int}$ are the effective conductance coefficients of the gel and intergel phases, respectively, calculated from the Nernst-Einstein relation as:

$$L_{i}^{g}=\frac{D_{i}^{g}C_{i}^{g}}{RT}$$

$$L_{i}^{int}=\frac{D_{i}^{s}C_{i}^{s}}{RT}$$

Where, $D_{i}^{g}$ and $D_{i}^{s}$ are the diffusion coefficients of the species i in the gel and solution phase, respectively, $C_{i}^{g}$ and $C_{i}^{s}$ are the concentrations of ions in the gel and solution phase, respectively, where i represents either co-ions (co) or counter-ions (ct). Their values are obtained from equations described below. R is the universal gas constant, and T is the temperature (K).

The gel phase and the intergel solution are assumed to be in local equilibrium. Then the ion concentration in the gel phase, $C_{i}^{g}$, is related to the ion concentration in the intergel

solution, $C_{i}^{s}$, by the Donnan equilibrium relation [10]:

$$\frac{(c_{+}^{g})^{\frac{1}{z_{+}}}}{{(c}_{-}^{g})^{\frac{1}{z_{-}}}}=K_{d}\frac{(c_{+})^{\frac{1}{z_{+}}}}{(c_{-})^{\frac{1}{z_{-}}}}$$

In the case of the membrane with high ion exchange capacity and binary electrolyte solution this relation may be approximated and written as follows:

$c_{-}^{g}=\frac{K_{d}}{Q_{g}}(c_{-}^{s})^{2}$, $c_{+}^{g}=Q_{g}+c_{-}^{g}$

where subscript “+” denotes the counterion (the membrane is assumed a cation-exchange one), “-“denotes the co-ion; $K_{d}$ is the Donnan constant, $Q_{g}$ is the ion exchange capacity of the gel phase (the concentration of charged fixed groups in the

gel); $Q_{g}$ is related to the ion exchange capacity of the membrane Q by the equation: $Q_{g}$= Q/*f_g_*.

Samples of membranes of known weight were converted into Na^+^ form by soaking in 1 M NaCl solution. After the membranes were washed with water they were put at equilibrium absorption in 0.1 M HCl during 24 h at 25 ˚C. They were finally placed in 1 M NaCl where the ion exchange capacities Q were determined by titration and expressed as the amount of sorbed H^+^ [21].

For a particular membrane, some of them could be considered as tabular values ($D_{i}^{s}$

is assumed the same as in free solution); the others should be estimated experimentally ($K_{d}$, $Q_{g}$, $D_{i}^{g}$, α). Homogeneous membranes have the volume fraction of intergel solution in the range of 0.05-0.15, in comparison to heterogeneous ones for which this parameter is between 0.2 and 0.3.

The co-ion ($C_{co}^{g}$) and counter-ion concentration ($C_{ct}^{g}$), at the membrane-feed interface (within the membrane) is calculated using Donnan equilibrium, assuming that the co-ion is SO_4_^2-^, and the counter-ion is Zn^2+^

$$C_{co}^{g}=C_{s}*\sqrt{\Gamma}exp(-asinh\frac{C_{fix}^{g}}{2C_{s}\sqrt{\Gamma}})$$

$$C_{ct}^{g}=\frac{-(z_{co}C_{co}^{g}+z_{fix}C_{fix}^{g})}{z_{ct}}$$

Where, $C_{s}$ is the solution concentration, $z_{fix}$ is the dimensionless signed charge and $C_{fix}^{g}$ the concentration of the fixed charges on the membrane, respectively, $z_{co}$ and $z_{ct}$ are the dimensionless signed charges of the co- and counterions, and Г is assumed to be 1 for an “ideal” Donnan model, meaning that both the bulk electrolyte and ions inside the membrane behave as ideal solutions, i.e., the activity coefficients of the solutions inside and outside the membrane are the same.

The diffusion coefficients (*D_i_*) in the gel phase are calculated by simultaneously solving equations for the ionic conductivity of the membrane (κ) and the salt coupled diffusion coefficient ($D_{s}^{m}$) obtained from experiments [11]:

$$k=\frac{F^{2}}{RT}(Z_{ct}^{2}D_{ct}^{g}C_{ct}^{g}+Z_{co}^{2}D_{co}^{g}C_{\mathrm{co}}^{g}）$$

$$D_{s}^{m}=\frac{D_{ct}^{g}D_{co}^{g}(z_{ct}^{2}z_{ct}^{g}+z_{co}^{2}C_{co}^{g})}{(z_{ct}^{2}D_{ct}^{g}C_{ct}^{g}+z_{co}^{2}D_{co}^{g}C_{co}^{g})}$$

Where κ is the membrane ionic conductivity, obtained from membrane specifications, F is the Faraday constant, $D_{co}^{g}$and $D_{ct}^{g}$ are the diffusion coefficients of the co- and counter ion respectively in the gel phase of the membrane.

**Electrochemical measurements**

To investigate the electrochemical stripping/plating behavior and cycling stability of the PS-Zn, bare Zn and the bioinspired PMCl-Zn electrode, symmetric cells were assembled into standard CR2032-type coin cells by using a glass fiber (Whatman, GF/A) separator and a 1 M ZnSO_4_ aqueous electrolyte. The hydrogen evolution polarization curves were collected by using the two-electrode linear sweep voltammetry (LSV) mode at a low scan rate of 5 mV s^-1^. The A.C. impedance spectra were recorded on the electrochemical workstation over a frequency range from 0.01 Hz to 10^5^ Hz at an AC voltage of 5 mV. The ionic conductivity was examined on powder pellets by using the alternating current (AC) impedance spectroscopy technique. The Zn^2+^ transference numbers were evaluated from the symmetric cells via EIS on the samples before and after chronoamperometry (CA) tests. For the full battery test, the electrodes were prepared by mixing commercial V_2_O_5_, acetylene black, and polyvinylidene fluoride (PVDF) at a weight ratio of 80%:10%:10%, the loading mass of V_2_O_5_ is about 1.7 mg cm^-2^. The mixture was dispersed in a small amount of methyl-2-pyrrolidinone (NMP) solvent by grinding in an agate mortar to achieve a stably homogeneous paste. The slurry was pasted on stainless steel foil and dried at 100 °C for 12 hours in a vacuum oven. The cells were galvanostatically charged/discharged over the voltage range of 0.3–1.5 V vs. Zn/Zn^2+^ at different current densities on Land CT2001A battery testers. The pouch-type batteries were assembled by sandwiching the electrolyte and the separator between the 3 cm × 5 cm V_2_O_5_ cathode and the bioinspired Zn@C anode and sealed by Al-plastic films. The dosage of the electrolyte was about 1.5 mL.

**DFT Computational Method**

DFT calculations were performed using spin-polarized density functional theory (DFT) framework as implemented in the Quantum-Espresso package. [12] The electron-ion and exchange-correlation interactions were described by ultrasoft pseudopotentials and the generalized gradient approximation (GGA) with Perdew-Burke-Ernzerhof (PBE) functional, respectively. [13-14] The Kohn-Sham (KS) orbitals and the charge density were represented using basis sets consisting of plane waves (PWs) up to a maximal kinetic energy of 50 Ry and 400 Ry, respectively. ClO_4_ group was incorporated into Zn-MOF units by absorbing on the active sites of Zn-linker and carbon chain, respectively. The long-range dispersion effect was considered using van der Waals correction in Grimme’s DFT-D3 scheme. [15] The deposition of Zn in the pure and ClO_4_ functionalized Zn-MOF units, respectively was stacked in the order of 002 direction. Monkhorst-Pack scheme was used to perform the integration in the Brilliouin zone with gamma for geometric optimization and 3×3×3 for electronic structure analysis. [16] All structures are optimized with convergence criteria of 1×10^-7^ eV for the energy and 1×10^-4^ eV/Å for the force. The adsorption energies of ClO_4_ in Zn-MOF and the binding energies Zn cluster on ClO_4_ functionalized MOF structures are calculated using the equation as follows:

*E*_adsorption_ (ClO_4_) = *E*_MOF-ClO4_ – *E*_MOF_ – *E*_ClO4_

*E*_bind_ (Zn cluster) = *E_Zn-_*_ClO4-MOF_ – *E* _ClO4-MOF_ – *E*_Zn cluster_

Where *E*_MOF-ClO4_ is the total energy of the ClO_4_ adsorbed in Zn-MOF structure, *E_Zn-_*_ClO4-MOF_ is the total energy of Zn cluster bound with ClO_4_ functionalized MOF structures, *E*_MOF_ is the energy of the Zn-MOF, *E*_Zn cluster_ is the energy of Zn cluster, and *E*_ClO4_ is the energy of ClO_4_.


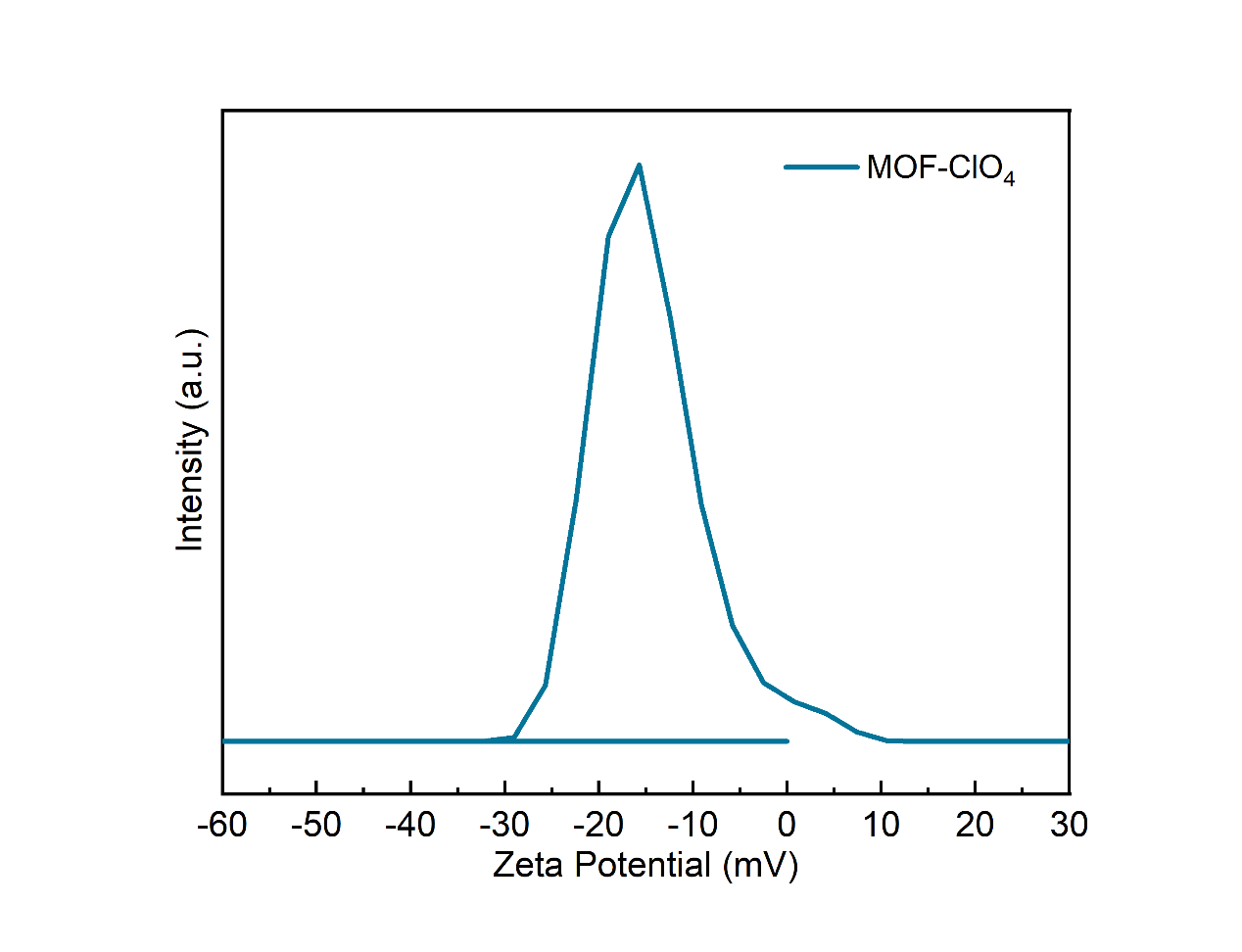


Figure S2. The Zeta potential of the MOF-ClO_4_


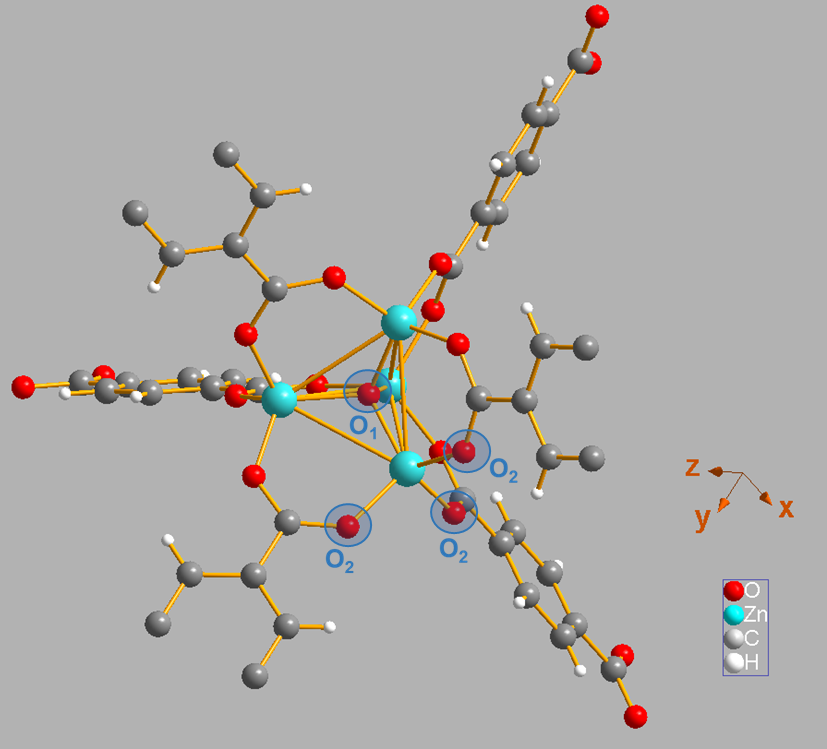


Figure S3. The ball-and-stick model of the MOF-5 sub-unit.

Through the SALE process, the cleavage of original Zn-O bonds in the MOF-5 occurred to form new Zn-O bonds to graft with -ClO_4_ groups. In pristine MOF-5, each Zn^2+^ is coordinated by one inorganic O atom (O_1_) and three O atoms in 1,4-terephthalic acid (O_2_). After grafting, the -ClO_4_ group could replace either the O_1_ or O_2_ site. DFT results show that the incorporation -ClO_4_ in MOF-5 at the linker O_2_ site is more energy favorable (-1.12 eV) than the O_1_ site connected with the carbon chains (-0.95 eV).


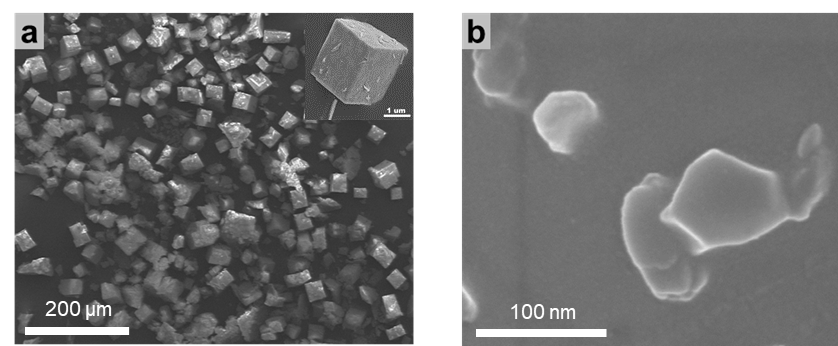


Figure S4. (a) The MOF-5 particles and (b) the MOF-ClO_4_ particles.


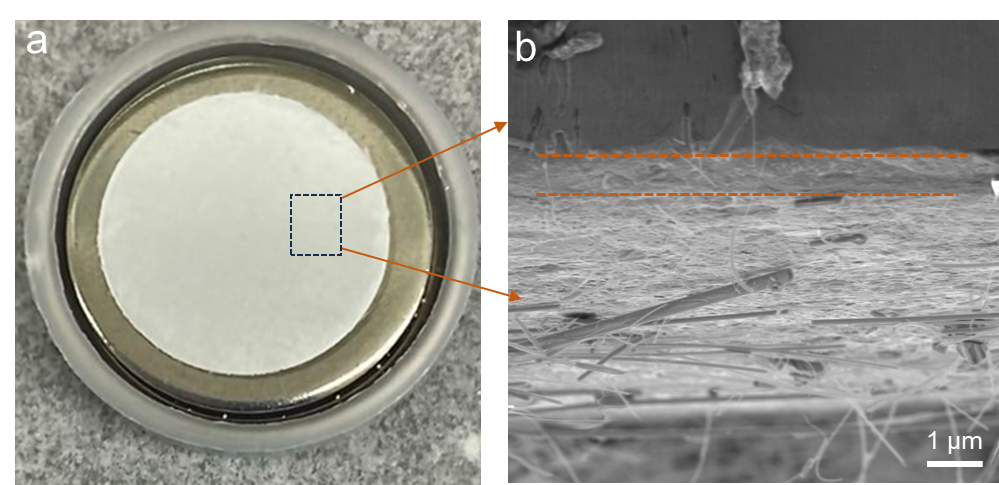


Figure S5. (a) The optical photo of PMCl-Zn electrode, (b) the cross-section SEM image of the thick of the PMCl fiber.


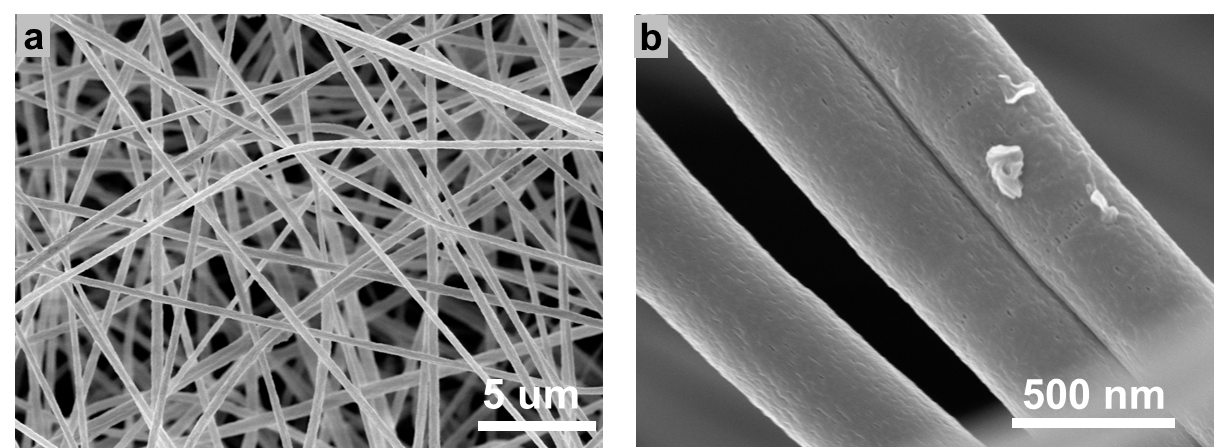


Figure S6. The SEM images of (a) PS fiber on the Zn surface and (b) the magnified PS fiber.


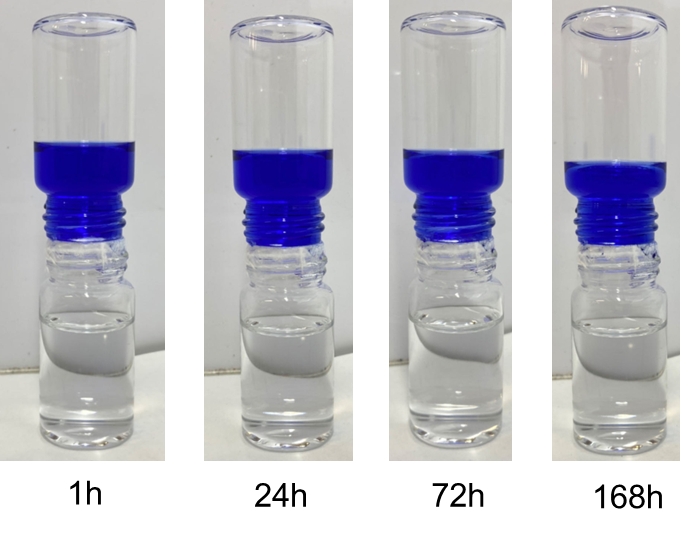


Figure S7. The water penetration experiment. The top chamber is H_2_O with blue ink as an indicator, and the bottom chamber is H_2_O, the PMCl membrane was used to separate the two chambers. Even after 7 days, the water still can’t penetrate the membrane to the bottom, as there is no color and water volume changes in the bottom.


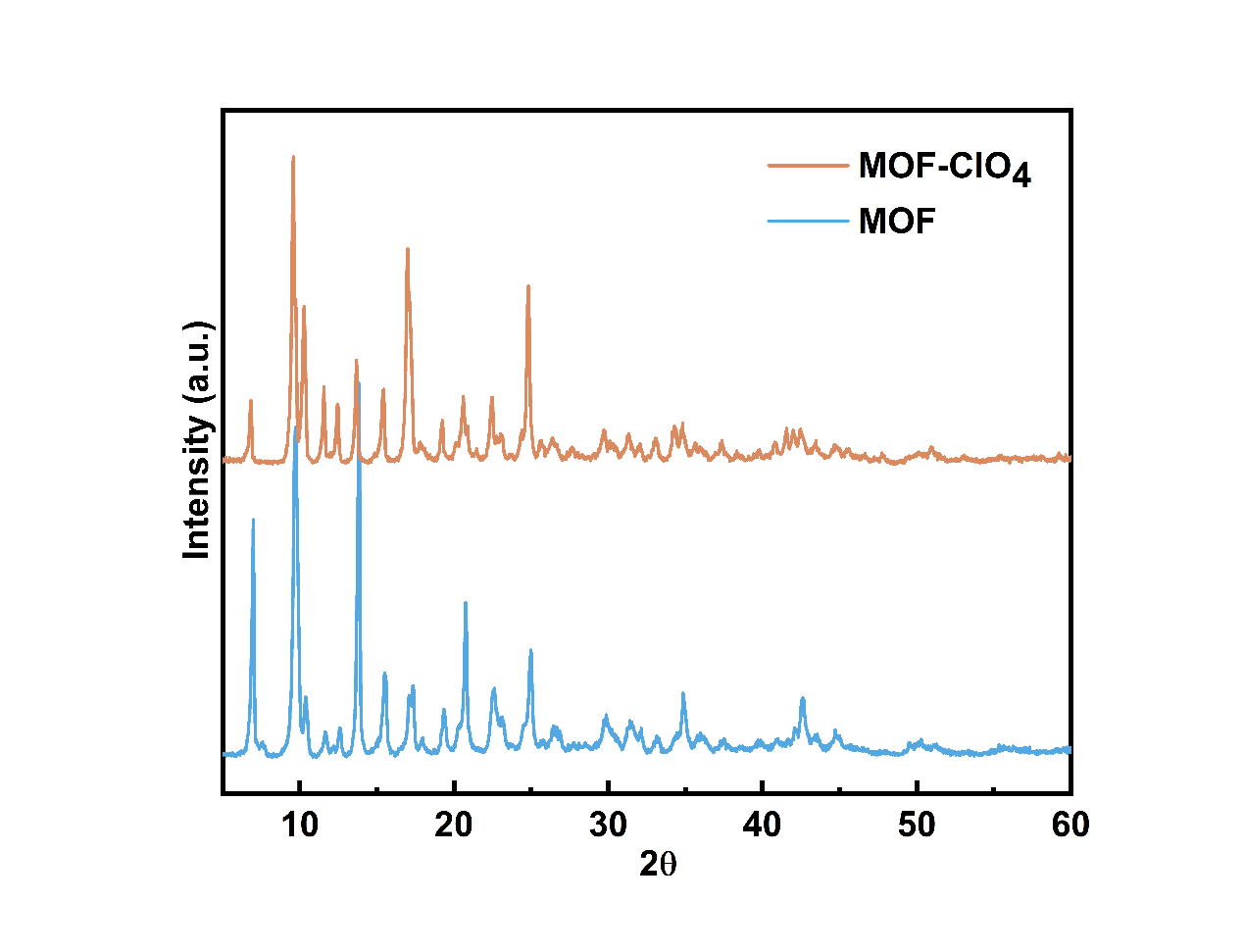


Figure S8. The XRD patterns of the MOF-ClO_4_ and MOF-5 particles.





Figure S9. Ionic conductance of the membrane as a function of electrolyte concentration.


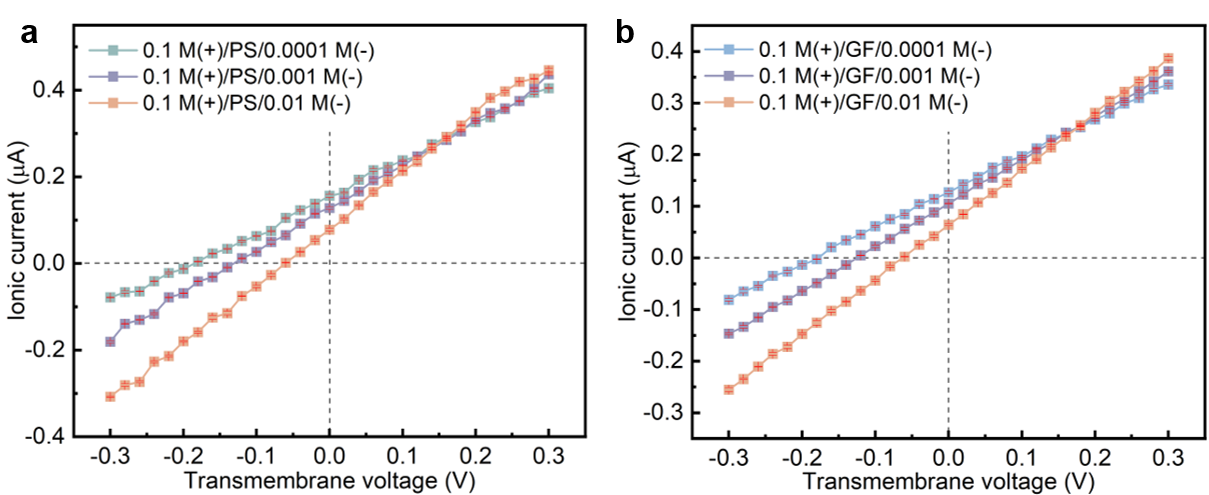


Figure S10. Drift-diffusion I–V curves of **a** PS and **b** GF separators measured in concentration gradient KCl electrolyte.

In comparison, the net current under the concentration gradient were, 4.78 μA (Δ=1000) and 4.34 μA (Δ=100) and 2.67 μA (Δ=10) for PMCl; 0.15 μA (Δ=1000) and 0.13 μA (Δ=100) and 0.075 μA (Δ=10) for PS (Figure S7a), and 0.12 μA (Δ=1000) and 0.1 μA (Δ=100) and 0.066 μA (Δ=10) for GF (Figure S7b), respectively. The higher positive net current means the stronger cations selectivity, which further proves the effect of MOF-ClO_4_ for realizing cation-sieving.





Figure S11. Cation transference number, t_+_, of PMCl, PS, and GF membranes under concentration gradients of Δ=10, 100 and 1000;





Figure S12. *I-V* curves of PMCl, PS, and GF membranes at 0.01 M, and the insert is the ionic rectification ratio, |I^+^|/|I^-^|. The ionic rectification ratio is defined as the ratio of the absolute current at +1 V (|I^+^|) to the one at -1 V (|I^-^|);


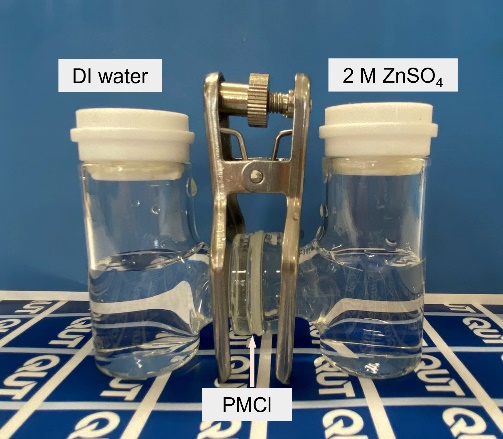


Figure S13. The H-shape device (inner diameter=15 mm) for the ion permeation test with 20 mL liquid at each side. One side is deionized water, and another side is the 1 M ZnSO_4_ solution, the membranes are mounted between the two chambers.


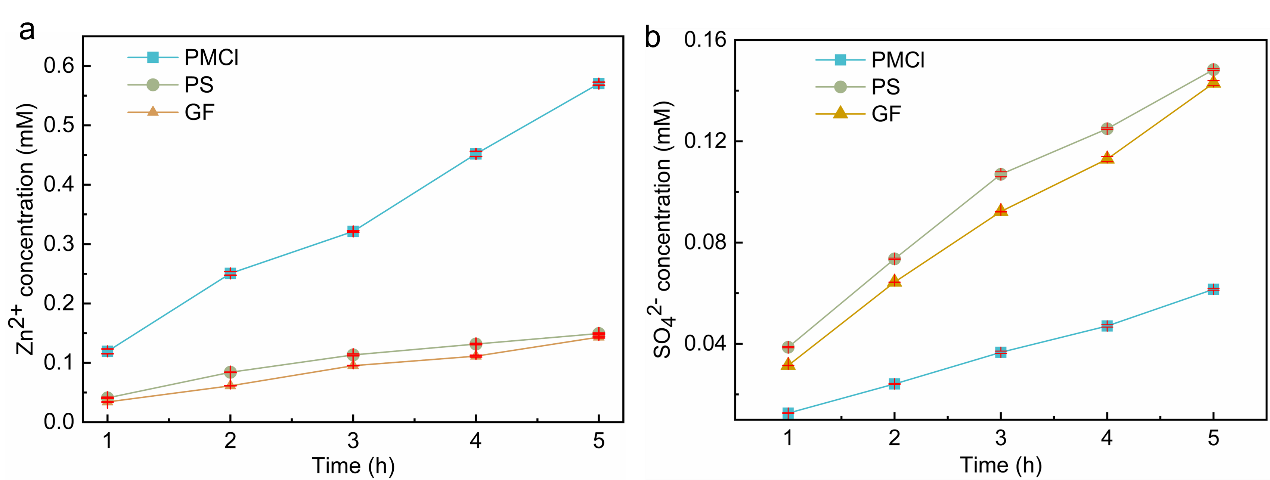


Figure S14. The concentration values for the **a** Zn^2+^ and **b** SO_4_^2-^ concentration after different penetration time.


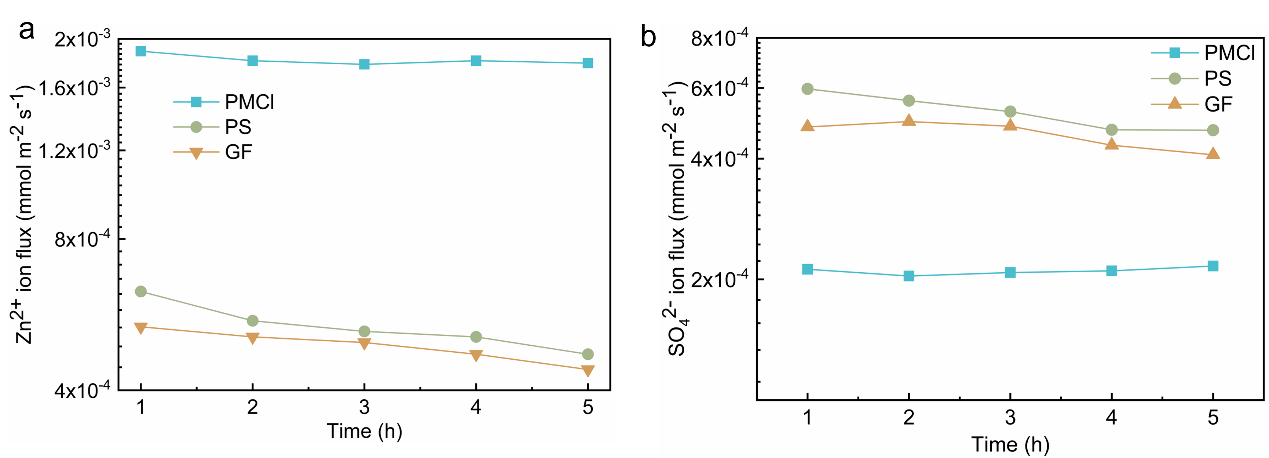


Figure S15. **a** Zn^2+^ ion flux and **b** SO_4_^2-^ ion flux of the three membranes determined experimentally with time.


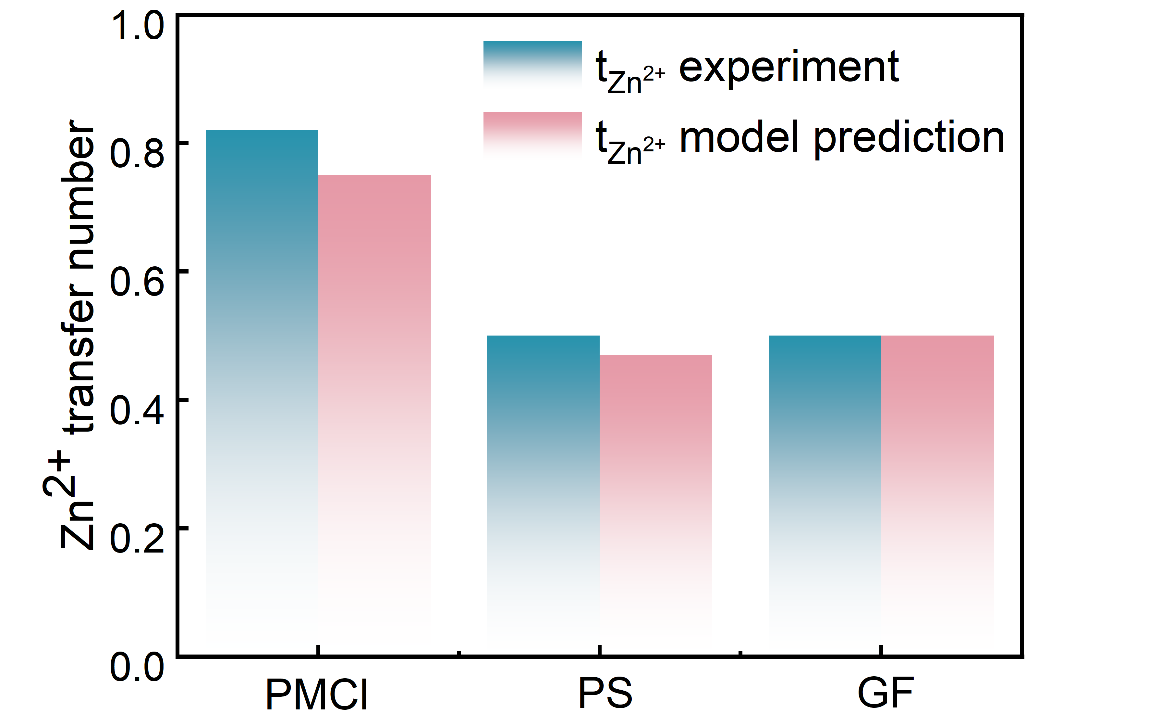


Figure S16. Zn^2+^ ion transfer numbers of the three membranes determined experimentally (blue) and via the mathematical model (red).


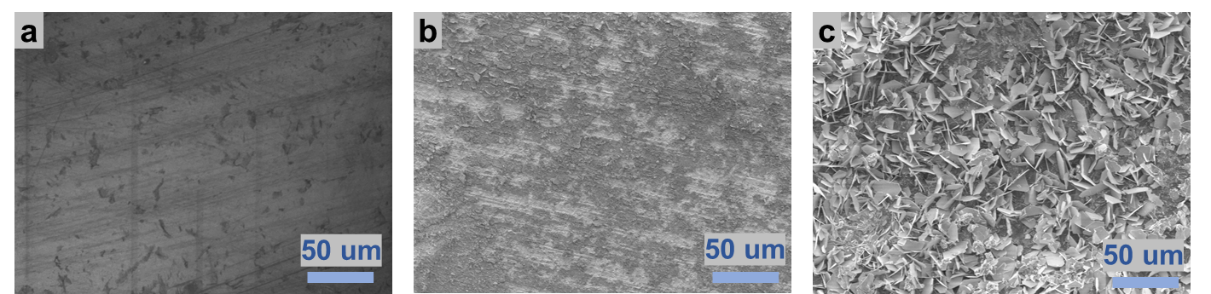


Figure S17. The SEM images of a PMCl-Zn, b PS-Zn and c bare Zn electrodes after soaking in the 1 M ZnSO_4_ electrolyte for 7 days.





Figure S18 Conductivity test of PMCl-Zn by blocking electrodes with an applied current of 10 mA, the PMCl-Zn electrode was sandwiched between two stainless steel current collectors. According to the formula of,

$$\boldsymbol{\rho}\mathbf{=}\frac{\boldsymbol{R*S}}{\boldsymbol{L}}=\frac{\boldsymbol{U*S}}{\boldsymbol{I*L}}$$

Where, L is thickness of the PMCl film; I is the applied current; S is area of the contact; U is the average voltage increase, so the resistivity of PMCl-Zn electrode is around 7.7×10^4^ Ω∙cm.


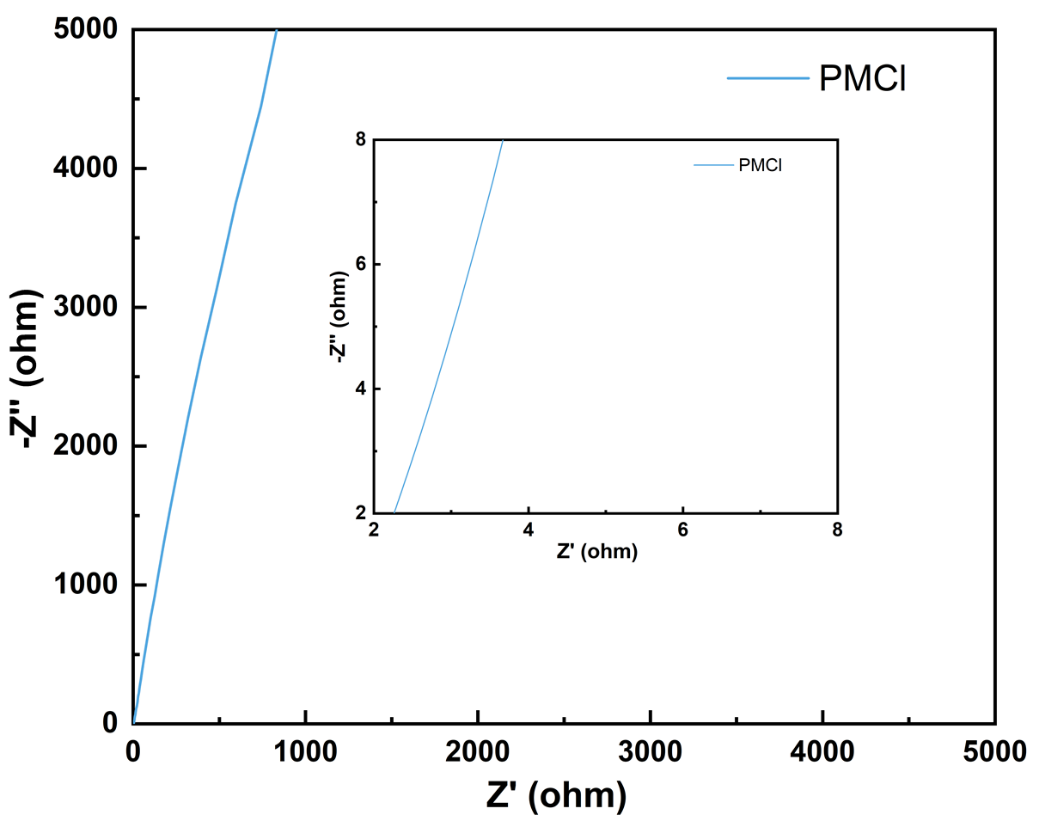


Figure S19. The ionic conductivity measurements of PMCl by using blocking electrodes. The PMCl membrane was pressed into a pellet with an average thickness of 2 μm and assembled into blocking cells to test. According to the following formula:

$$\boldsymbol{\delta}\mathbf{=}\frac{\boldsymbol{L}}{\boldsymbol{R}_{\boldsymbol{b}}\boldsymbol{S}}$$

Where *L* is the thickness of the carbon spheres, and *R_b_* and *S* represent the bulk resistance and the effective contact area, respectively.

For the R_b_ is 2.3 and the S is 1.1 cm^-2^, the ionic conductivity is ~1.26 mS cm^-2^.


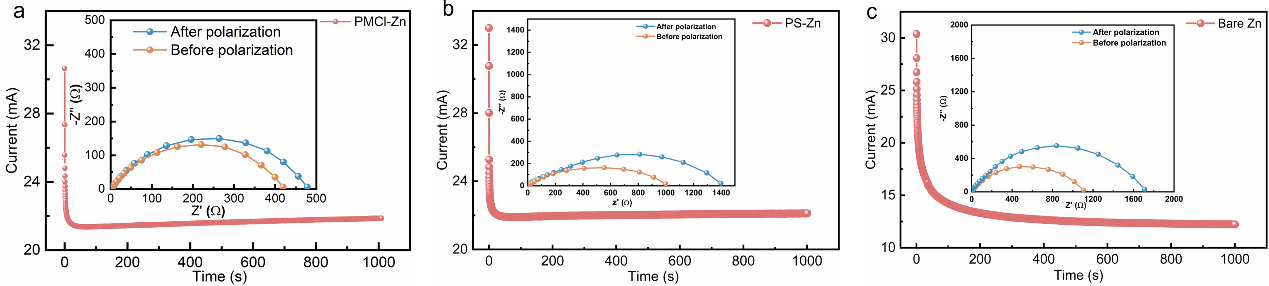


Figure S20. Current-time plots of (a) PMCl-Zn, (b) PS-Zn and (c) bare Zn symmetric cell after polarization at a constant potential (25 mV) for 3600 s. The insets are the impedance spectra before and after the polarization.

The transference number of Zn^2+^ ($t_{\mathrm{Zn}^{2+}}$) is determined by the following equation:

$$\boldsymbol{t}_{\boldsymbol{Zn}^{\boldsymbol{2+}}}\boldsymbol{=}\frac{\boldsymbol{I}_{\boldsymbol{s}}\boldsymbol{(\Delta V-}\boldsymbol{I}_{\boldsymbol{o}}\boldsymbol{R}_{\boldsymbol{o}}\boldsymbol{)}}{\boldsymbol{I}_{\boldsymbol{o}}\boldsymbol{(\Delta V-}\boldsymbol{I}_{\boldsymbol{s}}\boldsymbol{R}_{\boldsymbol{s}}\boldsymbol{)}}$$

where ΔV is the applied voltage; *I_0_* and *R_0_* are the initial current and resistance and *I_s_* and *R_s_* are the steady-state current and resistance, respectively.

Where the Δ*V* is 25 mV; For the PMCl-Zn cell, *I_0_* is 30.6 mA, *I_s_* is 21.8 mA, *R_o_* is 420 Ω and *R_s_* is 478 Ω, the calculated$t_{\mathrm{Zn}^{2+}}$for PMCl-Zn electrode is 0.88.

For the PS-Zn cell, *I_0_* is 33.1 mA, *I_s_* is 22.2 mA, *R_o_* is 988 Ω and *R_s_* is 1408 Ω, the calculated$t_{\mathrm{Zn}^{2+}}$for PS-Zn electrode is 0.69.

For the bare Zn cell, *I_0_* is 30.2 mA, *I_s_* is 12.2 mA, *R_o_* is 1105 Ω and *R_s_* is 1710 Ω, the calculated$t_{\mathrm{Zn}^{2+}}$for bare Zn electrode is 0.64.





Figure S21. The XRD peak intensity ratio for the (002) with (101) (denoted as *I_002_:I_101_*) and (100) (denoted as *I_002_:I_100_*) at 0.5 mA cm^-2^ and 2 mA cm^-2^.

The peak intensity ratio of the (002) to (101) (denoted as *I_002_:I_101_*) and (100) (denoted as *I_002_:I_100_*) could also be an indicator to show the orientation of the Zn deposition. The higher *I_002_:I_klh_* value means more horizontal (002)-dominant deposition. As shown in Figure S15, the PMCl-Zn electrode showed the highest (002) preferred orientation at different current densities, e.g., the *I_002_:I_100_* value reached 6.5 for PMCl-Zn but 3 for PS-Zn and 2.5 for bare Zn electrodes at 0.5 mA cm^-2^.


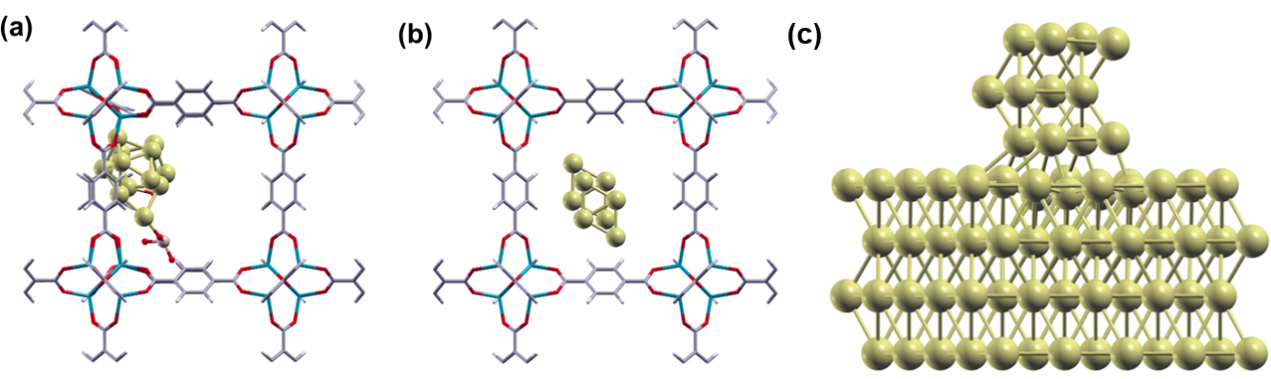


Figure S22. The DFT results for the (002) plane adsorption energy at (a) carbon chain (b) pure MOF-5 (c) bare Zn.

As we discussed before, the -ClO4 groups are likely to graft on both the carbon site and link site of MOF-5. The adsorption energies of Zn 002 cluster to MOF-ClO_4_ with ClO_4_ absorbed on the linker site, and MOF-ClO_4_ with ClO_4_ adsorbed on the carbon chain site are -4.47 eV and -1.10 eV respectively.


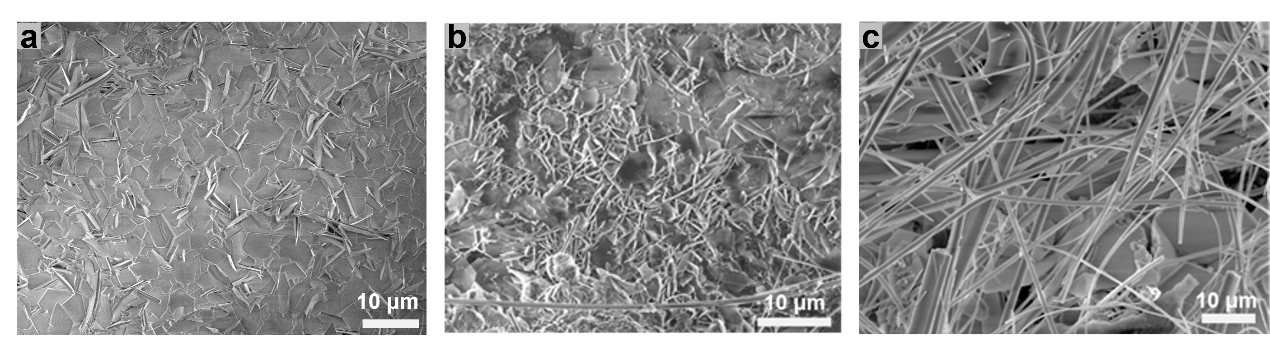


Figure S23. SEM images of a PMCl-Zn, b PS-Zn and c bare Zn electrodes after cycling at 0.5 mA cm^-2^_,_ 2 mAh cm^-2^.

While cycling at 0.5 mA cm^-2^/2 mAh cm^-2^, significant dendrites were observed on the bare Zn and PS-Zn electrodes as well, but the PMCl-Zn anode still maintained perfect (002) preferred orientation.


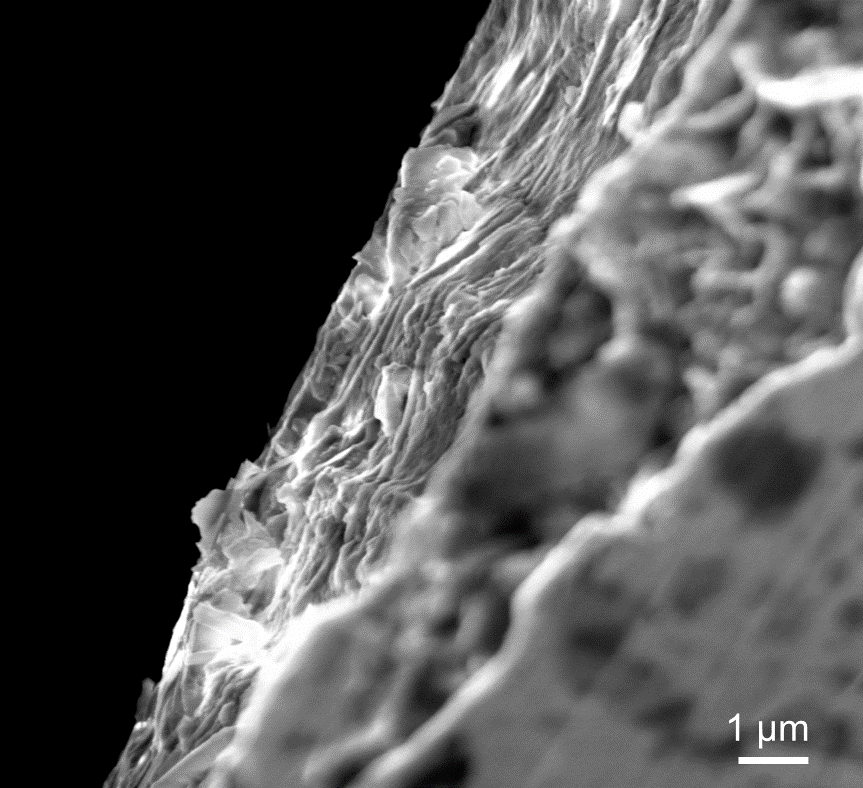


Figure S24. SEM cross-section image of the deposited Zn on the Zn anode surface and beneath the PMCl membrane with a horizontal direction.


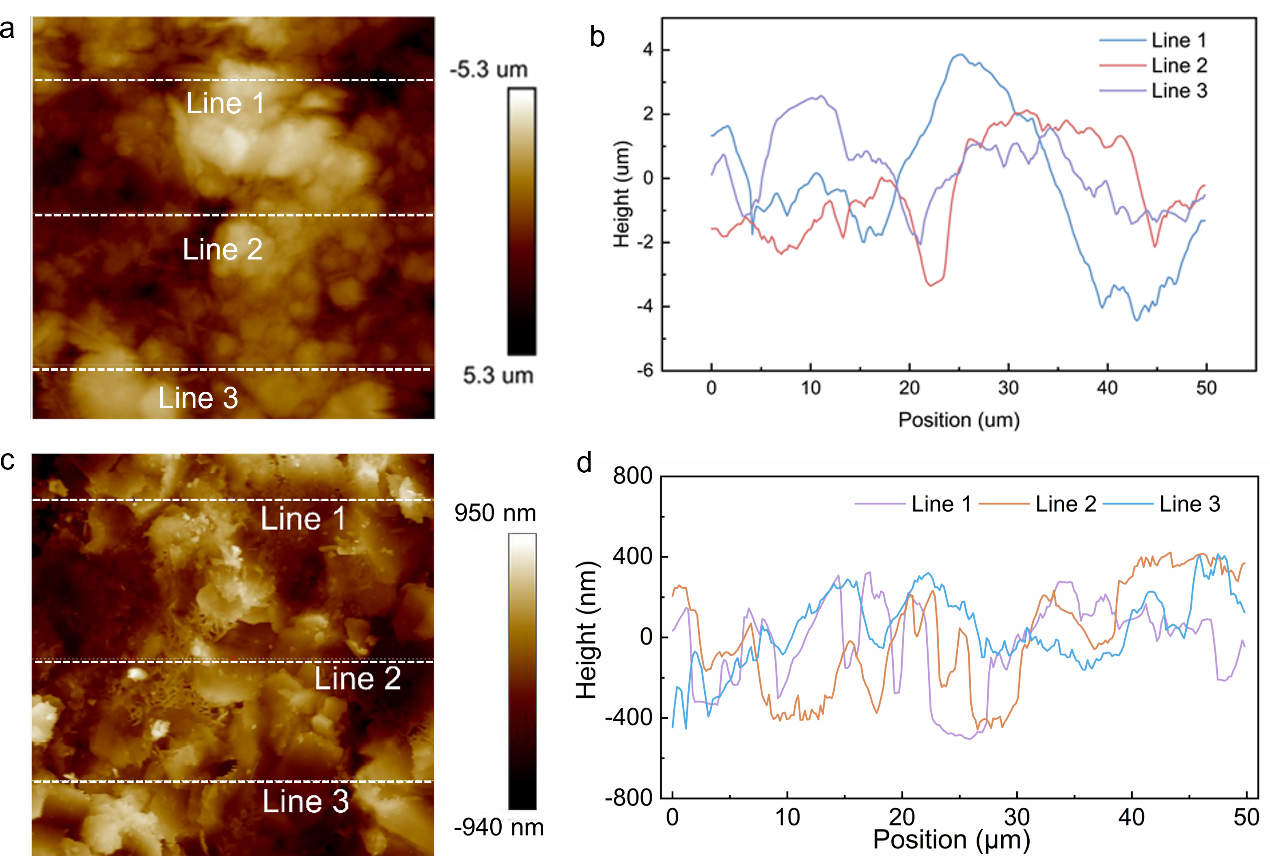


Figure S25. Corresponding vertical profiles along the indicated lines show surface roughness of a, b bare Zn and c, d PMCl-Zn electrodes.

The average height difference was less than 900 nm for the PMCl-Zn electrode, in contrast to more than 5 um for the bare Zn electrode.


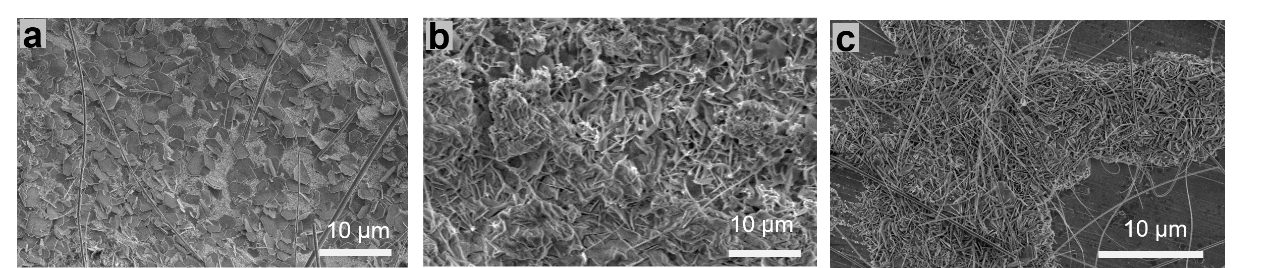


Figure S26. SEM images of a PMCl-Cu, b PS-Cu and c bare Cu electrodes after first Zn plating at 0.5 mA cm^-2^_,_ 2 mAh cm^-2^.





Figure S27. The XRD patterns of the PMCl-Zn, PS-Zn and bare Zn symmetric cells after cycling 50 cycles.

By comparing Figure S27 with Figure S8, we can conclude that the weak peaks in PMCl-Zn profiles at ~27°, 33°, 37° in Figure S27 should be from the signal of the MOF-ClO_4_.


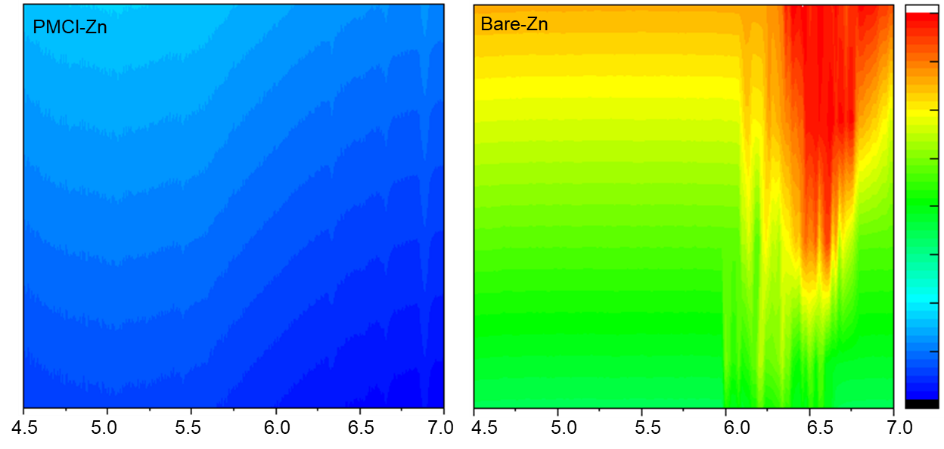


Figure S28. In-situ GC curves to evaluate the H_2_ generation during the Zn plating/stripping by using PMCl-Zn and bare Zn electrodes.


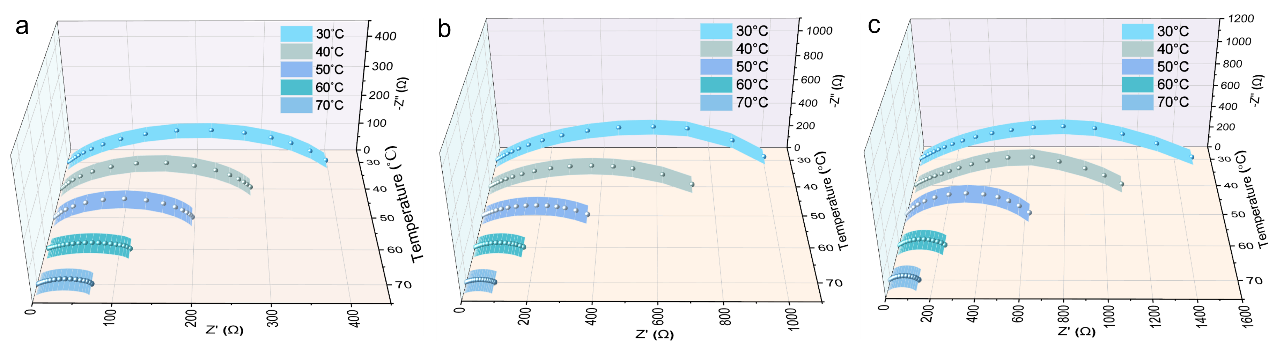


Figure S29. Nyquist patterns at different temperatures of the (a) PMCl-Zn (b) PS-Zn and (c) bare Zn anodes.

The desolvation process activation energy (*E_a_*) could be obtained by the Arrhenius equation:

$$\frac{1}{R_{ct}}=Aexp(-\frac{E_{a}}{RT})$$

Where R_ct_ is the interfacial resistance, *A* the frequency factor, *R* the gas constant, and *T* the absolute temperature. The R_ct_ value can be measured by the electrochemical impedance spectroscopy (EIS) at different temperatures. Figure S21 shows the fitted EIS curves of the three symmetrical cells at a temperature range 30˚C-80˚C.


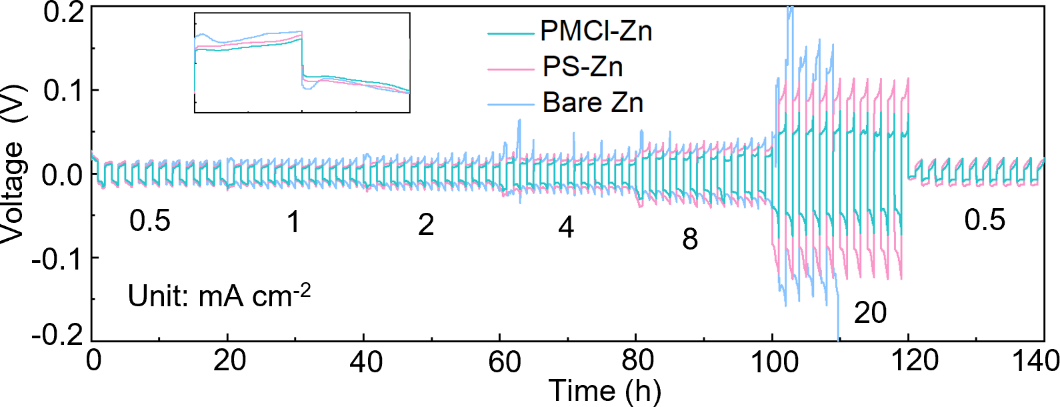


Figure S30. Rate performance of the Zn//Zn, PS-Zn//PS-Zn, and PMCl-Zn//PMCl-Zn symmetric cells tested at current densities from 0.5 to 20 mA cm^-2^.


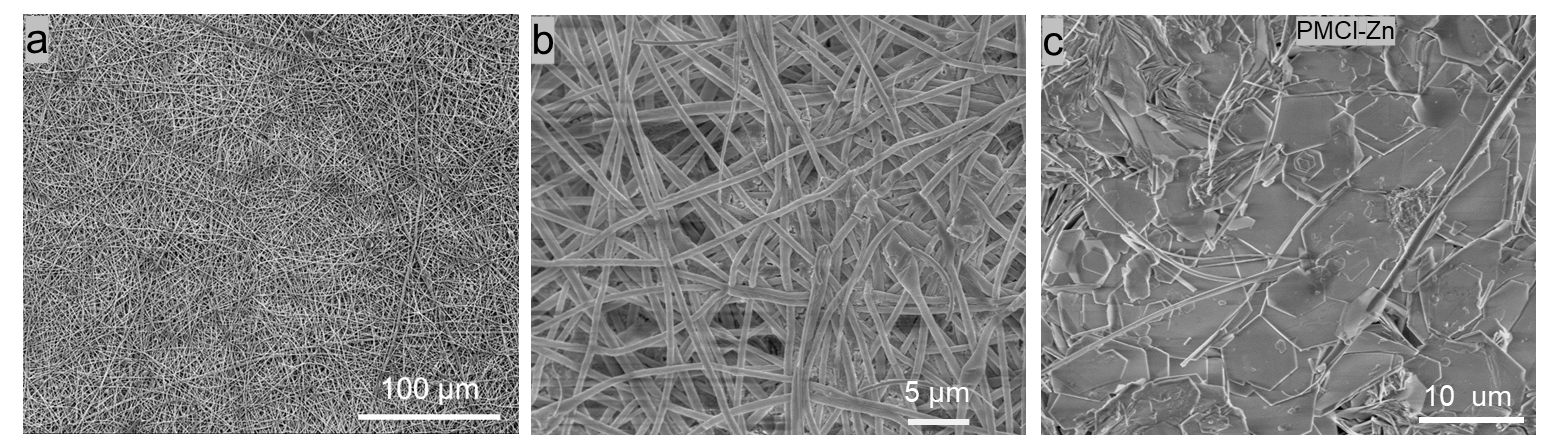


Figure S31. (a), (b) The SEM image of the PMCl-Zn surface after cycling for 5400 h at 10 mA cm^-2^. (c) The SEM image of the Zn surface after peeling off the PMCl membrane.

As shown in the SEM images, the PMCl membrane didn’t undergo physical damage after long-term Zn platting/striping, and no obvious Zn deposition formed on the PMCl membrane surface.


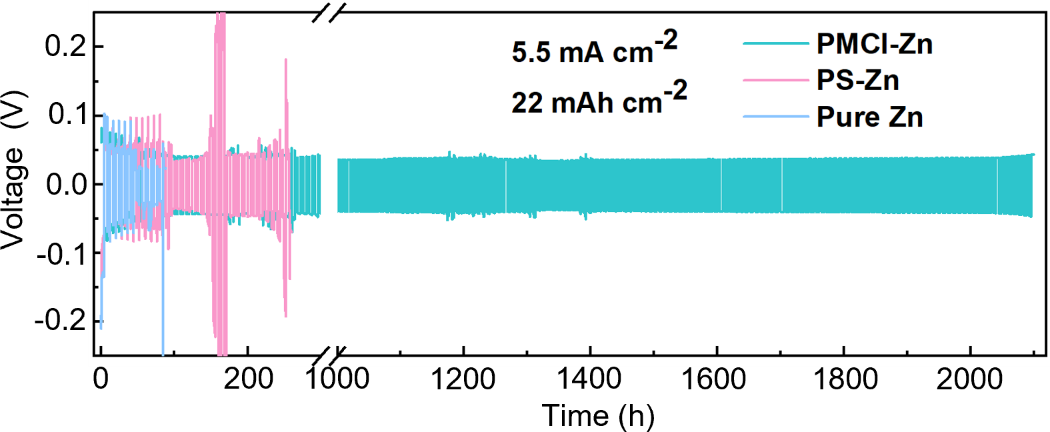


Figure S32. cycling performance at 5.5 mA cm^-2^ and 22 mAh cm^-2^;


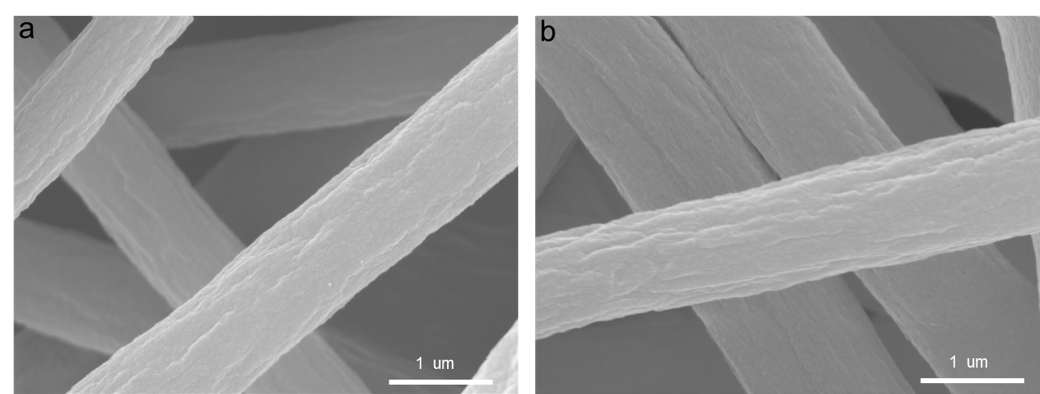


Figure S33 The SEM images of the PMCl fiber before (a) and after (b) the cycling test.





Figure S34. Galvanostatic charge/discharge (GCD) profiles of the Cu//Zn, Cu//PS-Zn and Cu //PMCl-Zn half-cells at 0.5 mA cm^-2^ and 0.5 mAh cm^-2^.


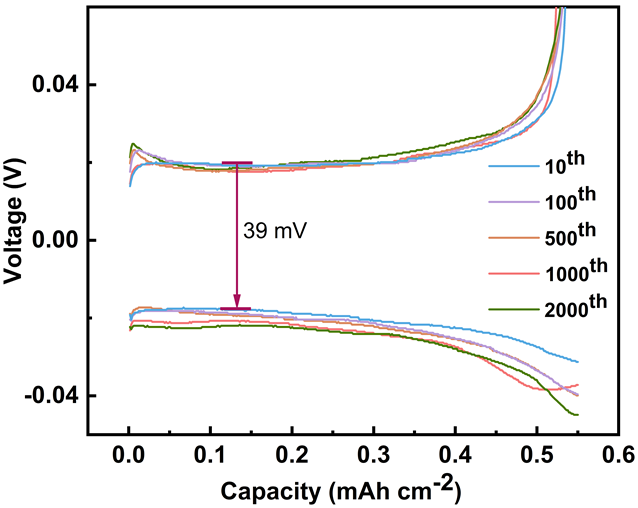


Figure S35. The magnified views of the 10^st^, 100^th^, 500^th^, 1000^th^, 2000^th^ hours for Cu//PMCl-Zn half-cell.


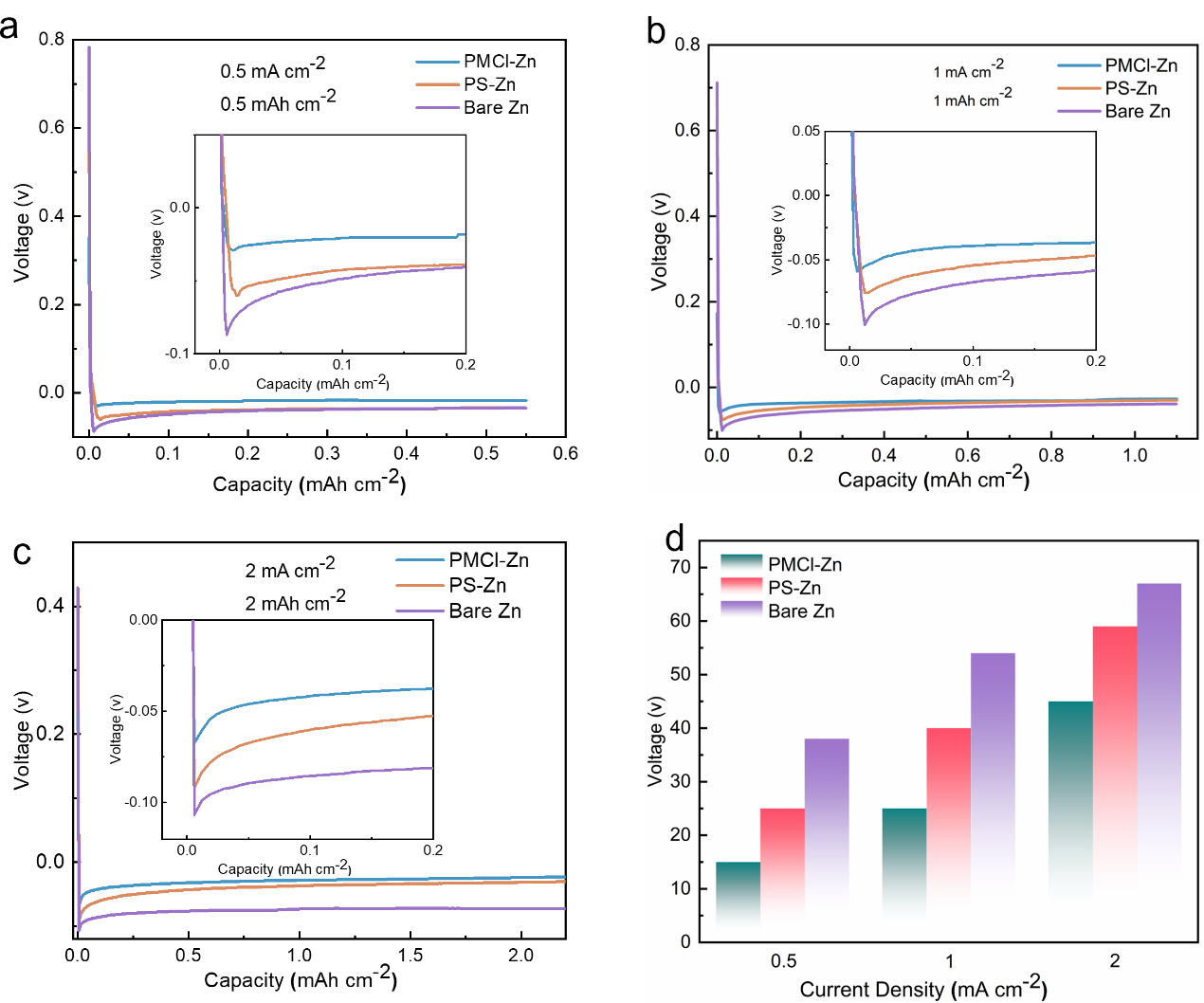


Figure S36. The nucleation overpotential of PMCl-Zn, PS-Zn and bare Zn electrodes at (a) 0.5 mA cm^-2^, (b) 1 mA cm^-2^ and (c) 2 mA cm^-2^, (d) the nucleation overpotential values at different current density.


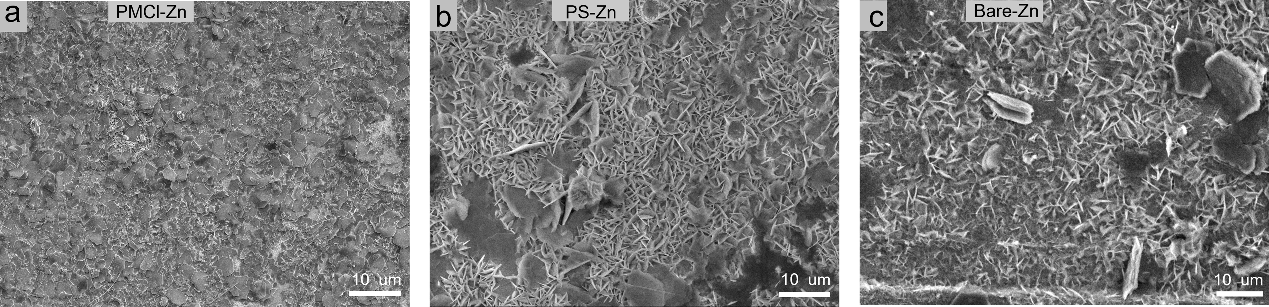


Figure S37. (a) post-testing Zn surface morphology of PMCl-Zn anode, (b) PS-Zn anode; and (c) bare Zn anode of a Zn-V_2_O_5_ pouch cell after cycling 500 times.


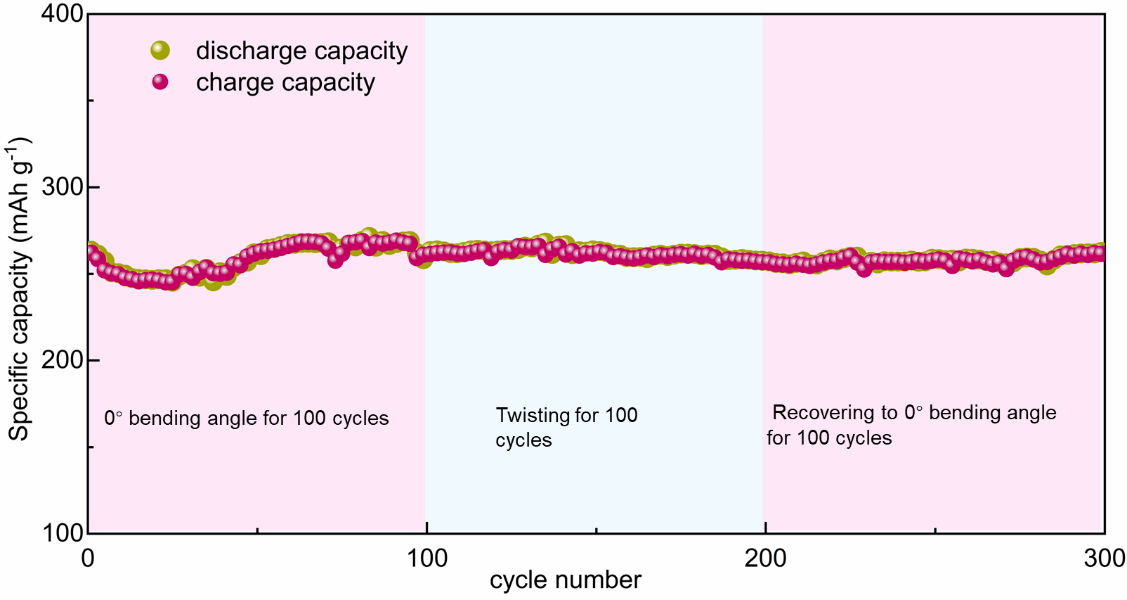


Figure S38 The cycling performance of the pouch cell tested at 0˚ angle again for 100 cycles, then twisting for 100 cycles, and recovering to 0˚ angle again for 100 cycles at 2 A g^-1^.

Table S1

Experimental details of zero-current voltage (E_m_), E_redox_, and real E_m_ values:

|  | γa (L) | γb (H) | E_m_ (mV) | E_redox_ (mV) | Real E_m_ (mV) | u^+^/u^-^ |
| --- | --- | --- | --- | --- | --- | --- |
|  | 0.902 | 0.782 | 305.6 | 156.8 | 148.8 | 11.5 |
| PMCl | 0.965 | 0.782 | 214.6 | 109.8 | 104.8 | 7.5 |
|  | 0.988 | 0.782 | 101.1 | 53.9 | 44.2 | 6.9 |
|  | 0.902 | 0.782 | 185.9 | 156.8 | 29.1 | 1.4 |
| PS | 0.965 | 0.782 | 122.8 | 109.8 | 13 | 1.21 |
|  | 0.988 | 0.782 | 61.5 | 53.9 | 7.6 | 1.3 |
|  | 0.902 | 0.782 | 180.2 | 156.8 | 23.4 | 1.3 |
| GF | 0.965 | 0.782 | 120.2 | 109.8 | 10.4 | 1.18 |
|  | 0.988 | 0.782 | 59.3 | 53.9 | 5.4 | 1.19 |

Table S2: Membrane properties

| Membrane properties | | | | | | | | | | | | |
| --- | --- | --- | --- | --- | --- | --- | --- | --- | --- | --- | --- | --- |
| Q_PMCl_  (meq cm^-3^)  0.93 | | Q_PS_  (meq cm^-3^)  0.98 | | | | Q_GF_  (meq cm^-3^)  1.01 | *w_u_*  PMCl  9.01 | *w_u_*  PS  6.14 | | *w_u_*  GF  4.55 | | x (thickness µm)  10 |
| Solution Properties | | | | | | | | | | | | |
| $C_{i}^{s}$ (M)  1 | $D_{{Zn}^{2+}}^{g}$  (10^-9^ m^2^ s^-1^)  0.71 | | | $D_{{Zn}^{2+}}^{int}$  (10^-9^ m^2^ s^-1^)  1.23 | | | $D_{{{SO}_{4}}^{2-}}^{g}$  (10^-9^ m^2^ s^-1^)  1.07 | | | $D_{{{SO}_{4}}^{2-}}^{int}$  (10^-9^ m^2^ s^-1^)  0.43 | | |
| Constants | | | | | | | | | | | | |
| R  J/mol/K  8.314 | | | T  K  298 | | | | $\rho_{w}$  g/L  998 | | | | $k_{d}$  0.1 | |
| Volume fractions | | | | | | | | | | | | |
| PMCl  *f_g_ f_int_*  0.9 0.1 | | | | | PS  *f_g_ f_int_*  0.86 0.14 | | | | GF  *f_g_ f_int_*  0.82 0.18 | | | |

Table S3

The comparation of the ion flux and ions electivity with the reported works:

| NO. |  | Ion flux | selectivity | Ref. |
| --- | --- | --- | --- | --- |
| 1. | Plasma membrane K^+^ ion channel KcsA | K^+^$1.67\times{10}^{-3}$ mol m^-2^ s^-2^ | K^+^/Na^+^ >1000 | Nature |
| 2 | Biomimetic KcsA channels | K^+^$2.62\times{10}^{-3}$ mmol m^-2^ s^-2^ | K^+^/Na^+^ =31 | Nat. Commun. |
| 3 | Biomimetic K^+^, Na^+^ channel | K^+^$3.6\times{10}^{-5}$ mmol m^-2^ s^-2^ | K^+^/Na^+^ (No)  K^+^/Mg^+^ =33 | J. Membr. Sci. |
| 4 | MOF-based biological ion channels | Li^+^, Na^+^, K^+^$1.6\times{10}^{-3}$ mmol m^-2^ s^-2^ | Li^+^/K^+^ =2.2  Li^+^/Na^+^=1.4 | Sci. Adv. |
| 5 | angstrom-sized GO-based ion channels | Na^+^, Cl^-^$2.78\times{10}^{-4}$ mmol m^-2^ s^-2^ | Cl^+^/Na^+^<1 | Science |
| 6 | angstrom-sized GO-based ion channels | Li^+^, Na^+^, K^+^${10}^{-3}$ mmol m^-2^ s^-2^ | K^+^/Na^+^<1  K^+^/Li ^+^<1 | Nat. Nanotechnol. |
| 7 | Hydrous manganese  oxide (HMO) phosphates ions selective membrane | phosphates ions $2.17\times{10}^{-4}$ mmol m^-2^ s^-2^ | H_2_PO_4_^-^/Cl^-^=20 | Nat. Nanotechnol. |
| 8 | polyethylene terephthalate (PET) Hostaphan® films | K^+^$5.5\times{10}^{-4}$ mol m^-2^ s^-2^ | K^+^/Mg^+^ =20 | Adv. Funct. Mater. |
| 9 | cross-linked graphene oxide membrane | K^+^$1.6\times{10}^{-3}$ mol m^-2^ s^-2^ | K^+^/Mg^+^ =7 | J. Membr. Sci. |

Table S4

The diffraction intensity of the randomly oriented sample:

| Intensity |  | 002 100 101  0.5 mA cm^-2^ | | | 002 100 101  1 mA cm^-2^ | | |
| --- | --- | --- | --- | --- | --- | --- | --- |
| I_0_  PMCl-Zn  PS-Zn  Pure Zn |  | 53  6722  2914  2925 | 40  1029  958  1181 | 100  5421  4337  5518 | 53  3392  938  850 | 40  937  436  757 | 100  1872  5466  5934 |

Table S5

The comparation of the cycling performance with the reported works:

| NO. | Current density  (mA cm^-2^) | | Capacity  (mAh cm^-2^) | Time  (h) | Reference |
| --- | --- | --- | --- | --- | --- |
| 1  2  3  4  5  6  7  8  9  10  11  12  13  14  15  16  17  18  19  20  21  22 | | 5  10  1  7.5  10  1  10  0.5  5  2  1  10  5.5  5  5  4  5  10  1  10  1  10 | 0.5  10  1  7.5  20  2  1  1  1  2  1  20  22  10  5  0.5  5  10  1  2.5  1  1 | 1300  500  4000  400  360  1600  160  5000  1750  1000  6000  5400  2400  800  1300  1900  350  1400  1400  1600  2000  600 | 25  26  27  28  29  30  31  32  33  34  Our work  Our work  Our work  35  36  37  38  39  40  41  42  43 |

**References**

1. S. S. Kaye, A. Dailly, O. M. Yaghi, J. R. Long, Impact of preparation and handling on the hydrogen storage properties of Zn_4_O(1,4-benzenedicarboxylate)_3_ (MOF-5) J. Am. Chem. Soc. **2007**, 129,14176.
2. W. Xin, J. Fu, Y. Qian, L. Fu, X. Kong, T. Ben, L. Jiang, L. Wen, Biomimetic KcsA channels with ultra-selective K^+^ transport for monovalent ion sieving, Nat. Commun. 2022, 13, 1701.
3. M. Porozhnyy, P. Huguet, M. Cretin, E. Safronova, V. Nikonenko, Mathematical modeling of transport properties of proton-exchange membranes containing immobilized nanoparticles. Int. J. Hydrogen Energy 2016, 41, 15605–15614.
4. B. Zhang, H. Gao, C. Xiao, X. Tong, Y. Chen, The trade-off between membrane permselectivity and conductivity: A percolation simulation of mass transport. J. Memb. Sci. 2020, 597, 117751.
5. V. Nikonenko, Modelling of ion transport in electromembrane systems: Impacts of membrane bulk and surface heterogeneity. Appl. Sci. 2018, 9.
6. X. Tong, Y. Wei, Fundamental studies of a new series of anion exchange membranes: Membrane preparation and characterization. J. Memb. Sci. 2001, 190, 159–166.
7. N. P. Gnusin, V. I. Zabolotsky, V.V. Nikonenko, A.I. Meshechkov. Development of the principle of general conductance to the description of transport phenomena in disperse systems, Zhurnal Fiz. Khimii. Rus J Phys Chem 1980, 54, 1518e22.
8. G. S. Gohil, V. K. Shahi, R. Rangarajan, Comparative studies on electrochemical characterization of homogeneous and heterogeneous type of ion-exchange membranes. J. Memb. Sci. 2004, 240, 211–219.
9. D. Ariono, Khoiruddin, Subagjo, I. G. Wenten, Heterogeneous structure and its effect on properties and electrochemical behavior of ion-exchange membrane. Mater. Res. Express 2017, 4.
10. M. Porozhnyy, P. Huguet, M. Cretin, E. Safronova, V. Nikonenko, Mathematical modeling of transport properties of proton-exchange membranes containing immobilized nanoparticles, Int. J. Hydrogen energy. 2016, 41, 15605-15614.
11. J. Kamcev, D. R. Paul, G. S. Manning, B. D. Freeman, Ion diffusion coefficients in ion exchange membranes: significance of counterion condensation. Macromolecules 2018, 51, 5519–5529.
12. Giannozzi, P., Baroni, S., Bonini, N., et al. Quantum ESPRESSO: a modular and open-source software project for quantum simulations of materials. J. Phys.: Condens. Matter 2009, 21, 395502.
13. Vanderbilt, D. Soft self-consistent pseudopotential in a generalized eigenvalue formalism. Phys. Rev. B 1990, 41, 7892-7895.
14. Perdew, J. P., Burke, K. & Ernzerhof, M. Generalized gradient approximation made simple. Phys. Rev. Lett. 1996, 77, 3865-3868.
15. Grimme, S, Antony, J., Ehrlich, S., Krieg, S. A consistent and accurate ab initio parametrization of density functional dispersion correction (DFT-D) for the 94 elements H-Pu. J. Chem. Phys. 2010, 132, 154104.
16. Monkhorst, H. J. & Pack, J. D. Special points for Brillouin-zone integrations. Phys. Rev. B 1976, 13, 5188-5192.
17. S. Y. Noskov, S. Bernèche, B. Roux, Control of ion selectivity in potassium channels by electrostatic and dynamic properties of carbonyl ligands. Nature 2004, 431, 830–834.
18. E. S. Hatakeyama, C. J. Gabriel, B. R. Wiesenauer, J. L. Lohr, M. Zhou, R. D. Noble, D. L. Gin, Water filtration performance of a lyotropic liquid crystal polymer membrane with uniform, sub-1-nm pores, J. Membr. Sci. 2011, 366, 62–72.
19. H. Zhang, J. Zhou, Y. Hu, P. Wang, R. Ou, L. Jiang, J. Z. Liu,B. D. Freeman, A. J. Hill, H, Wang, Ultrafast selective transport of alkali metal ions in metal organic frameworks with subnanometer pores, 2018, Sci. Adv. 4, eaaq0066.
20. R. K. Joshi, P. Carbon, F. C. Wang, V. G. Kravets, Y. Su, I. V. Grigorieva, H. A. Wu, A. K. Geim, R. R. Nair, Precise and ultrafast molecular sieving through graphene oxide membranes. Science 2014, 343, 752–754.
21. J. Abraham, K. S. Vasu, C. D. Williams, K. Gopinadhan, Y. Su, C. T. Cherian, J. Dix, E. Prestat, S. J. Haigh, I. V. Grigorieva, P. Carbone, A. K. Geim, R. R. Nair, Tunable sieving of ions using graphene oxide membranes. Nat. Nanotechnol. 2017, 12, 1222–1228.
22. A. Iddya, P. Zarzycki, R. Kingsbury, C. Khor, S. Ma, J. Wang, I. Wheeldon, Z. J. Ren, E. M. V. Hoek, D. Jassby, A reverse-selective ion exchange membrane for the selective transport of phosphate via an outer sphere complexation-diffusion pathway, Nat. Nanotechnol. 2017, 12, 546–550.
23. Q. Wen, D. Yan, F. Liu, M. Wang, Y. Ling, P. Wang, P. Kluth, D. Schauries, C. Trautmann, P. Apel, W. Guo, G. Xiao, J. Liu, J. Xue, Y. Wang, Highly selective ionic transport through subnanometer pores in polymer films. Adv. Funct. Mater. 2016, 26, 5796–5803.
24. Z. Jia, Y. Wang, W. Shi, J. Wang, Diamines cross-linked graphene oxide free-standing membranes for ion dialysis separation, J. Membr. Sci. 2016, 520, 139–144.
25. H. Yu, Y. Chen, W. Wei, X. Ji, L. Chen, A functional organic zinc-chelate formation with nanoscaled granular structure enabling long-term and dendrite-free Zn anodes, ACS Nano 2022, 16, 9736–9747.
26. M. Zhao, J. Rong, F. Huo, Y. Lv, B. Yue, Y. Xiao, Y. Chen, G. Hou, J. Qiu, S. Chen, Semi-immobilized Ionic Liquid Regulator with Fast Kinetics towards Highly Stable Zinc Anode under -35 °C to 60 °C, Adv. Mater. 2022, 34, 2203153.
27. J. Zhu, W. Deng, N. Yang, X. Xu, C. Huang, Y. Zhou, M. Zhang, X. Yuan, J. Hu, C. Li, R. Li, Biomolecular regulation of zinc deposition to achieve ultra-long life and high-rate Zn metal anodes, Small 2022, 18, 2202509.
28. Y. Hao, D. Feng, L. Hou, T. Li, Y. Jiao, P. Wu, Gel electrolyte constructing Zn (002) deposition crystal plane toward highly stable Zn anode, Adv. Sci. 2022, 9, 2104832.
29. Y. Lv, M. Zhao, Y. Du, Y. Kang, Y. Xiao, S. Chen, Engineering a self-adaptive electric double layer on both electrodes for high-performance zinc metal batteries, Energy Environ. Sci. 2022,15, 4748-4760.
30. Y. Zhao, M. Ouyang, Y. Wang, R. Qin, H. Zhang, W. Pan, D. Y. C. Leung, B. Wu, X. Liu, N. P. Brandon, J. Xuan, F. Pan, H. Wang, Biomimetic lipid-bilayer anode protection for long lifetime aqueous zinc-metal batteries, Adv. Funct. Mater. 2022, 2203019.
31. K. Zhao, G. Fan, J. Liu, F. Liu, J. Li, X. Zhou, Y. Ni, M. Yu, Y. Zhang, H. Su, Q. Liu, F. Cheng, Boosting the kinetics and stability of Zn anodes in aqueous electrolytes with supramolecular cyclodextrin additives, J. Am. Chem. Soc. 2022 144 (25), 11129-11137.
32. X. Zhang, J. Li, D. Liu, M. Liu, T. Zhou, K. Qi, L. Shi, Y. Zhu, Y. Qian, Ultra-long-life and highly reversible Zn metal anodes enabled by a desolvation and deanionization interface layer, Energy Environ. Sci. 2021, 14, 3120.
33. Z. Zhao, R. Wang, C. Peng, W. Chen, T. Wu, B. Hu, W. Weng, Y. Yao, J. Zeng, Z. Chen, P. Liu, Y. Liu, G Li, J. Guo, H. Lu, Z. Guo, Horizontally arranged zinc platelet electrodeposits modulated by fluorinated covalent organic framework film for high-rate and durable aqueous zinc ion batteries, Nat Commun. 2021, 12, 6606.
34. H. Jia, M. Qiu, C. Lan, H. Liu, M. Dirican, S. Fu, X. Zhang, Advanced zinc anode with nitrogen-doping interface induced by plasma surface treatment, Adv. Sci. 2021, 2103952.
35. Y. Chu, S. Zhang, S. Wu, Z. Hu, G. Cui, J. Luo, In situ built interphase with high interface energy and fast kinetics for high performance Zn metal anodes, Energy Environ. Sci. 2021, 14, 3609.
36. Z. Huang, Z. Li, Y. Wang, J. Cong, X. Wu, X. Song, Y. Ma, H. Xiang, Y. Huang, Regulating Zn (002) deposition towards long cycle life for Zn metal batteries, ACS Energy Lett. 2023, 8, 372–380.
37. W. Zhang, Y. Dai, R. Chen, Z. Xu, J. Li, W. Zong, H. Li, Z. Li, Z. Zhang, J. Zhu, F. Guo, X. Gao, Z. Du, J. Chen, T. Wang, G. He, I. Parkin, Highly reversible zinc metal anode in a dilute aqueous electrolyte enabled by a pH buffer additive, Angew. Chem. Int. Ed. 2022, e202212695.
38. M. Qiu, P. Sun, Y. Wang, L. Ma, C. Zhi, W. Mai, Anion-trap engineering toward remarkable crystallographic reorientation and efficient cation migration of Zn ion batteries, Angew. Chem. Int. Ed. 2022, e202210979.
39. X. Zhang, J. Li, K. Qi, Y. Yang, D. Liu, T. Wang, S. Liang, B. Lu, Y. Zhu, J. Zhou, An Ion-sieving Janus separator toward planar electrodeposition for deeply rechargeable Zn-metal anodes.
40. Y. Su, B. Liu, Q. Zhang, J. Peng, C. Wei, S. Li, W. Li, Z. Xue, X. Yang, J. Sun, Printing-scalable Ti_3_C_2_Tx MXene-decorated Janus separator with expedited Zn^2+^ flux toward stabilized Zn anodes, Adv. Funct. Mater. 2022, 32, 2204306.
41. Y. Liang, D. Ma, N. Zhao, Y. Wang, M. Yang, J. Ruan, G. Yang, H. Mi, C. He, P. Zhang, Novel concept of separator design: efficient ions transport modulator enabled by dual-interface engineering toward ultra-stable Zn metal anodes, Adv. Funct. Mater. 2022, 32, 2112936.
42. J. Zhu, Z. Bie, X. Cai1, Z. Jiao, Z. Wang, J. Tao, W. Song, H. Fan, Molecular sieve electrolyte membrane enables separator-free zinc batteries with ultralong cycle life, Adv. Mater. 2022, 34, 2207209.
43. C. Li, Z. Sun, T. Yang, L. Yu, N. Wei, Z. Tian, J. Cai, J. Lv, Y. Shao, M. H. Rümmeli, J. Sun, Z. Liu, Directly grown vertical graphene carpets as Janus separators toward stabilized Zn metal anodes, Mater. 2020, 32, 2003425.
